# Competitive interactions facilitate resistance development against antimicrobials

**DOI:** 10.1128/aem.01155-23

**Published:** 2023-10-11

**Authors:** Luka Svet, Ilse Parijs, Simon Isphording, Bram Lories, Kathleen Marchal, Hans P. Steenackers

**Affiliations:** 1 Department of Microbial and Molecular Systems, Centre of Microbial and Plant Genetics (CMPG), Leuven, Belgium; 2 Department of Plant Biotechnology and Bioinformatics, Data Integration and Biological Networks, UGent, Technologiepark 15, Gent, Belgium; INRS Armand-Frappier Sante Biotechnologie Research Centre, Laval, Quebec, Canada

**Keywords:** microbial ecology, microbial communities, biofilms, drug resistance evolution, intermicrobial interactions

## Abstract

While the evolution of antimicrobial resistance is well studied in free-living bacteria, information on resistance development in dense and diverse biofilm communities is largely lacking. Therefore, we explored how the social interactions in a duo-species biofilm composed of the brewery isolates *Pseudomonas rhodesiae* and *Raoultella terrigena* influence the adaptation to the broad-spectrum antimicrobial sulfathiazole. Previously, we showed that the competition between these brewery isolates enhances the antimicrobial tolerance of *P. rhodesiae*. Here, we found that this enhanced tolerance in duo-species biofilms is associated with a strongly increased antimicrobial resistance development in *P. rhodesiae*. Whereas *P. rhodesiae* was not able to evolve resistance against sulfathiazole in monospecies conditions, it rapidly evolved resistance in the majority of the duo-species communities. Although the initial presence of *R. terrigena* was thus required for *P. rhodesiae* to acquire resistance, the resistance mechanisms did not depend on the presence of *R. terrigena*. Whole genome sequencing of resistant *P. rhodesiae* clones showed no clear mutational hot spots. This indicates that the acquired resistance phenotype depends on complex interactions between low-frequency mutations in the genetic background of the strains. We hypothesize that the increased tolerance in duo-species conditions promotes resistance by enhancing the selection of partially resistant mutants and opening up novel evolutionary trajectories that enable such genetic interactions. This hypothesis is reinforced by experimentally excluding potential effects of increased initial population size, enhanced mutation rate, and horizontal gene transfer. Altogether, our observations suggest that the community mode of life and the social interactions therein strongly affect the accessible evolutionary pathways toward antimicrobial resistance.

**IMPORTANCE:**

Antimicrobial resistance is one of the most studied bacterial properties due to its enormous clinical and industrial relevance; however, most research focuses on resistance development of a single species in isolation. In the present study, we showed that resistance evolution of brewery isolates can differ greatly between single- and mixed-species conditions. Specifically, we observed that the development of antimicrobial resistance in certain species can be significantly enhanced in co-culture as compared to the single-species conditions. Overall, the current study emphasizes the need of considering the within bacterial interactions in microbial communities when evaluating antimicrobial treatments and resistance evolution.

## INTRODUCTION

The evolution of antimicrobial resistance has been extensively studied. However, most research focused on microbes cultivated in shaking liquid cultures (1–4). In such conditions, microbes adapt to a free-living (“planktonic”) way of life (5, 6). This planktonic lifestyle significantly differs from the way that microbes most commonly grow in natural, industrial, or clinical settings: in dense, structured, and heterogeneous biofilms. Inside these biofilm communities, microbes are encased in a self-produced slime layer, comprised of extracellular polymeric substances (7–10). Due to their persistence and high tolerance toward antimicrobial treatment (11–16), biofilms are often a source of resilient contaminations or chronic infections (17). Biofilm formation not only provides a temporary and condition-dependent tolerance against antimicrobial treatment, but there are also indications that it can accelerate antimicrobial resistance development. Indeed, (i) the heterogeneous environment supports diverse subpopulations on which selective forces can act independently (10, 18–20), and (ii) the high-density and prolonged cell-to-cell contact results in a high degree of horizontal gene transfer (21–23). As opposed to tolerance, the generated resistance can generally be retained in other conditions that are not necessarily associated with biofilm formation.

In nature, biofilms often consist of different strains and species that strongly interact with each other (10, 24). It has been well established that these inter-species social interactions can further enhance the tolerance of bacterial communities against antimicrobial treatment in a variety of ways (16, 24, 25). It is, however, largely unknown how social interactions in mixed-species communities shape resistance development. While most of the focus has been placed on horizontal gene transfer (26, 27), interspecies interactions could potentially also influence the emergence of novel resistant mutants via effects on population size (28) or mutation rate (29). In addition, social interactions could impact the selection of these resistant mutants by altering the advantage and cost of resistant mutants compared to their susceptible counterparts (30). We have recently proposed a theoretic framework summarizing these potential impacts of interspecies social behavior on the different aspects of resistance development in reference (31). Since the evolution of antimicrobial resistance has become one of the biggest health issues of the 21st century (32, 33), it is paramount to better understand how the natural way of life of bacteria, in dense and diverse biofilms, alters resistance development.

The brewery environment is one of the areas where antimicrobial resistance is of increasing concern (34, 35). The resistance of contaminating biofilms against commonly used sanitizers and disinfectants is increasing (36–38), which results in difficulties in disinfecting food-contact surfaces and environments. In a previous study, we characterized the social interactions and their impact on antimicrobial tolerance in an array of biofilms isolated from the brewery environment (16). In the current study, we built on these data and explored how social interactions between brewery biofilm isolates affect resistance development against the broad-spectrum antimicrobial sulfathiazole, which we identified as one of the most effective compounds against brewery isolates in a prior inhibitor screening (16). We specifically focused on a duo-species biofilm composed of the isolates *Pseudomonas rhodesiae* and *Raoultella terrigena*, members of two bacterial families that are most commonly encountered in brewery biofilms (39, 40). Considering that similar social interactions likely occur in many microbial communities, including communities containing pathogens (31), the conclusions of this study are expected to have broader implications.

## MATERIALS AND METHODS

### Definition of a repeat

Measurements performed on biologically distinct samples (e.g., microbial cells originating from different bacterial colonies) which are presumed to show biological variation are regarded as biological repeats, while technical repeats are repeated measurements performed on the same sample. Independent repeats are measurements performed on biological repeats at minimally two different timepoints.

### Sampling and identification of brewery isolates

Fifty-eight samples were taken from four different breweries in Belgium as described previously (16). The *Pseudomonas* and *Raoultella* isolates from sample 3.8 were further identified at the species level, based on analysis of their 16S rRNA genomic region and whole genome similarity (41). Briefly, a minute volume originating from the preserved overnight cultures of each species recovered from the brewery samples was used to initiate a new overnight culture to obtain sufficient material for whole genome sequencing. The DNA was extracted using the DNeasy Blood & Tissue Kit (Qiagen, Hilden, Germany) in accordance with the manufacturer’s instructions, and the whole genome, sequenced as described below. The 16S rRNA region was extracted from the genomic data and compared to the database of available 16S rRNA genomic regions accessible online at EzBioCloud. In addition, to further validate the identification, the whole genome similarity to several *Pseudomonas* and *Raoultella* genomes, respectively, was determined by calculating the average nucleotide identity value (41). These analyses were performed using the Geneious Prime 2023.1.2 software package (42).

### Growth of mono- and duo-species biofilms

Mono- and duo-species biofilms were grown as described previously (16). Specifically, overnight cultures of the different species separately were grown in 5 mL of plate count broth (5 g/L peptone, 2.5 g/L yeast extract, and 1 g/L glucose) in glass tubes in shaking conditions (200 RPM) at 25°C for 18 hours. Afterwards, mono- and duo-species biofilms were set up in 1/20 tryptic soy broth (TSB) (1.5 g/L) by inoculating 1,000 cells/mL of each species in flat-bottom, non-binding 96-well plates (Greiner Bio-One International, Kremsmünster, Austria). Each biological repeat (i.e., with microbial cells originating from different bacterial colonies) consisted of three technical repeats. To minimize cross-contamination, spillover, and evaporation of the medium, the wells were covered with a gas-permeable seal and enclosed with a plastic lid (Greiner Bio-One International, Kremsmünster, Austria). The bacterial communities were then grown for 4 days at 25°C in non-shaking conditions. Subsequently, the amount of biofilm formed at the bottom of the wells was quantified by colony-forming unit (CFU) counting. Hereto, planktonic cells were first removed by siphoning the liquid lying above the biofilms using a pipette. Their quantity was estimated via optical density measurement at 595 nm using the multimode reader. Next, 120 µL of phosphate-buffered saline [PBS: 8.8 g/L NaCl, 0.39 g/L KH_2_PO_4_, and 1.24 g/L K_2_HPO_4_ (pH 7.4)] was added to each biofilm-containing well, and afterwards, the biofilm cells were abraded off the bottom of the wells by a pipette tip. To disperse potential aggregates of biofilm cells, the liquid was vigorously pipetted up and down 10 times. Finally, the obtained suspension was serially diluted and plated out on plate count agar (PCA: 5 g/L peptone, 2.5 g/L yeast extract, 1 g/L glucose, and 15 g/L agar). The plates were then incubated for 2 days at 25°C. The colonies in plates that contained duo-species biofilms were discerned based on colony morphology (43, 44). If the growth dynamics of biofilms were determined, the microbial communities were plated out after the first, second, third, and fourth day of biofilm growth.

### Tolerance assay

To quantify the tolerance in mono- and duo-species communities toward the preventive sulfathiazole treatment, sulfathiazole (dissolved in dimethyl sulfoxide [DMSO]) from the previously prepared stock solution of 50 g/L was added to the growth medium used to inoculate the biofilm assay described above in order to obtain a final concentration of 100 µM. To account for the potential effects of DMSO on bacterial growth, the same volume of DMSO only was added as a control to untreated communities. The number of biofilm or planktonic cells was determined both in the presence and absence of sulfathiazole as described above. In order to determine the monospecies tolerance of strains evolved in the duo-species community (see below), the day 36 sample of the duo-species treated and untreated biofilms stored in the −80°C freezer was plated out, and colonies of *P. rhodesiae* and *R. terrigena* were selected and afterwards stored at −80°C. Those cultures were then also used to initiate the tolerance assay, employing the same procedure. Tolerance to the antimicrobial was defined according to the formulas below:


$$tolerance\left({species}_{i},mono\right)=\frac{growthoftreatedbacteria\left({species}_{i},mono\right)}{growthofuntreatedbacteria\left({species}_{i},mono\right)}$$

$$tolerance\left({species}_{i},multi\right)=\frac{growthoftreatedbacteria\left({species}_{i},multi\right)}{growthofuntreatedbacteria\left({species}_{i},multi\right)}$$

### Measuring the extracellular polymeric substance production

The production of extracellular polymeric substances (EPS) was quantified by employing the crystal violet assay in the Calgary biofilm device (Innovotech, Edmonton, Canada) (45). In brief, mono- and duo-species biofilms were set up in the presence or absence of sulfathiazole in three biological repeats as described above. The 96-well plates were then covered with a plastic lid with 96 pegs and grown for 4 days at 25°C in non-shaking conditions. Afterwards, each peg was washed with 200 µL of PBS and stained for 30 minutes with the same volume of crystal violet solution (0.1%, wt/vol; solvent: 1:1:18, vol/vol, isopropanol/methanol/PBS). Next, each peg was washed again using 200 µL of distilled water and left until completely dry (approximately 30 minutes). Finally, the pegs were destained with acetic acid (30%, vol/vol, 200 µL per peg), and the EPS, quantified by measuring the optical density at 570 nm using the Synergy Mx Microplate Reader (BioTek, Winooski, US.).

### Evolution of mono- and duo-species biofilms

A serial transfer evolution experiment was performed using the biofilm setup described above (see Fig. S1 for a schematic overview). Mono- and duo-species biofilms of *P. rhodesiae* and *R. terrigena* were evolved over a period of 80 days with or without sulfathiazole treatment. Twenty biological repeats were established for each condition, resulting in 120 biofilms. Within each repeat, the same overnight culture originating from a single bacterial colony was used to inoculate the different conditions, i.e., initial mono- or duo-species biofilms in the presence or absence of sulfathiazole; thus, 40 different overnight cultures were used in total (20 for each species employed). In the absence of sulfathiazole, an equivalent volume of the sulfathiazole solvent (DMSO) was added. Each species was inoculated in a density of approximately 1000 cells/mL. The bacterial communities were then incubated, and the biofilms, grown at 25°C. After 4 days, the planktonic cells were removed, and the biofilms at the bottom surface of the wells were abraded by a pipette tip and suspended in 120 µL of PBS. The isolated biofilm cells were then diluted in freshly prepared 1/20 TSB containing 100 µM sulfathiazole or an equivalent volume of DMSO. Since it has previously been shown that inoculum density can affect biofilm spatial structuring (46), we kept the initial inoculum size of control and treated lineages in the same order of magnitude by diluting control lineages 40,000 times and treated lineages 400 times, resulting in approximately 1,000 cells/mL in each condition after the first transfer. We kept the dilution factors constant over the course of the experiment to be able to compare the extinction rates between the treated mono- and duo-culture populations. After each cycle, 100 µL of the biofilm suspended in PBS was diluted in 100 µL of 50% glycerol and preserved at −80°C.

The quantity of planktonic cells above the biofilms was measured via optical density at 595 nm, and the number of viable and culturable biofilm cells at the bottom of 96-well plates was quantified every 8 days (starting on day 4 and ending on day 76) as described previously.

### Classification of social interactions

To characterize the ecological interplay within the biofilm communities, interactions between the species were classified based on the ratio of the number of cells in the mono- and duo-species biofilm. Interactions were designated as cooperative in case the biofilm growth of all constituent species in a duo-species microbial community was higher than their respective growth in monospecies biofilms (47). Contrarily, the interactions were deemed competitive if at least one of the species in duo-species community experienced a decreased biofilm growth as compared to its corresponding growth in a monospecies biofilm. In case when the biofilm growth of one of the constituent species in the duo-species community increased while the growth of the second species remained unaffected in comparison to their respective monospecies biofilm growths, the interactions were characterized as commensal.

### Estimating the mutation rate

To estimate the mutation rate in mono- and duo-species biofilms, a fluctuation assay (48) was adapted for the use with biofilm cultures. Specifically, overnight cultures of *P. rhodesiae* and *R. terrigena* were grown separately in plate count broth (in shaking conditions (200 RPM) at 25°C for 18 hours. Afterwards, 15 parallel monospecies biofilms were set up in 1/20 TSB in flat-bottom, non-binding 96-well plates (Greiner Bio-One International, Kremsmünster, Austria), and 15 duo-species biofilm communities were set up in 1/20 TSB in 24-well plates of the same manufacturer. Both mono- and duo-species biofilms were inoculated with 1,000 *P*. *rhodesiae* cells/mL, and in the case of duo-species biofilms, *R. terrigena* was added at a density of 1,000 cells/mL. To minimize cross-contamination, spillover, and evaporation of the medium, the wells were covered with a gas-permeable seal and enclosed with a plastic lid (Greiner Bio-One International, Kremsmünster, Austria). The bacterial communities were then grown for 3 days at 25°C in non-shaking conditions in the absence of antimicrobial. Subsequently, planktonic cells were removed from the microbial communities by siphoning the liquid above the biofilms using a pipette. Two hundred microliters of phosphate-buffered saline were added to each biofilm-containing well, and afterwards, the biofilm cells were abraded off the bottom of the wells by a pipette tip. To disperse the potential aggregates of biofilm cells to individual cells, the liquid was vigorously pipetted up and down 10 times. Finally, the biofilm cells were plated out in duplicate on non-selective PCA plates and selective PCA plates supplemented with 10 mg/L of gentamicin (2.5× MIC of *P. rhodesiae*). For non-selective plates, biofilm cells were first serially diluted, while serial dilution of the cultures was not performed for selective plates. Instead, 50% of the total cells were plated out (100 µL). Non-selective growth plates (three per specific condition) were used to estimate the total bacterial population size, while selective plates (12 per specific condition) were used to quantify the number of mutants formed. After 2 days of incubation at 25°C, the number of colonies was quantified. Differences in colony morphology allowed us to differentiate between *P. rhodesiae* and *R. terrigena* during CFU counting. Based on these results, the maximum likelihood estimate of m, the expected number of mutations per culture, under the Luria-Delbrück model that allows correction for low plating efficiency was calculated using the R package rSalvador v1.9 (49). The mutation rate per cell was obtained by dividing the number of mutations per culture by the total number of cells in the culture in question.

### Whole genome sequencing of starter and evolved biofilm strains

The genomic DNA of ancestral *P. rhodesiae* and *R. terrigena* strains and evolved clonal pools was isolated via the DNeasy Blood and Tissue Kit (Qiagen, Hilden, Germany) and sequenced using Illumina HiSeq4000 technology with PE150 bp reads. The evolved clonal pools consisted of 10 clones selected from a single sample on day 36 of the evolution experiment. The 10 selected clones were pooled, grown overnight, and subsequently subjected to sequencing. The selected *P. rhodesiae* clones were all shown to be sulfathiazole resistant (see below) and thus hypothesized to carry the adaptive variants resulting in sulfathiazole resistance. As a control, pools of 10 clones selected from the duo-species populations that evolved in the absence of sulfathiazole were also sequenced.

Additionally, with the purpose of obtaining data of sufficient quality to be able to *de novo* construct the ancestral genome of *P. rhodesiae* (as no reference was available), we performed another round of sequencing on a parental strain of *P. rhodesiae* using a combination of the PacBio Sequel II (mean subread length >11 kbp) and DNBSEQ platform (PE150 bp reads). The *de novo* assembly to reconstruct the ancestral strain genome as the reference for variant calling proceeded according to the Trycycler pipeline (50) employing the default parameters, with Polypolish (51) and POLCA (52) for short-read polishing of the assembly. The annotation of the resulting genome was performed using Prokka v1.14.6 (53). Afterwards, the genome was deposited at the GenBank of the National Center for Biotechnology Information (54) under the accession numbers of CP125985 (plasmid) and CP125986 (chromosome).

To identify variants that originated during evolution, we mapped the reads of the evolved samples of *P. rhodesiae* against the assembled reference. Variant and indel calling was performed using Snippy (55), BactSNP (56), and Geneious (42), employing the default settings. Variants with a frequency of 10% or more were selected for further scrutiny via literature research. This lenient threshold allowed us to identify resistance-conferring mutations that competed with other mutations and did not completely sweep the population. Furthermore, the *de novo* assemblies of the reads of the evolved strains of *Pseudomonas* that did not align to the reference genome were evaluated for traces of horizontal gene transfer that were potentially missed during the previous variant calling procedure. Briefly, the unaligned reads were *de novo* assembled using SPAdes (57) with the assemblies not satisfying the length (≥ 500 bp) and coverage (≥ 2) requirement then being discarded. Thus obtained assemblies were then screened against the NCBI database of *Raoultella* nucleotide and protein sequences employing blastn and blastp(58).

The sequencing data of these evolved samples were likewise deposited to the NCBI data repository and are accessible under the BioProject PRJNA972615 or the NCBI SRA accession numbers of SRR25440063, SRR25440064, SRR25440065, SRR25440066, SRR25440067, and SRR25440068.

## RESULTS

### The presence of competing *Raoultella strains* commonly results in enhanced tolerance of *Pseudomonas* toward antimicrobial treatment

Our prior research showed that the competition between a *Raoultella* and a *Pseudomonas* strain, co-isolated from contaminating biofilms in breweries*,* enhanced the tolerance of *Pseudomonas* toward sulfathiazole, a broad-spectrum antimicrobial that inhibited the majority of the examined brewery isolates (16, 59, 60). Tolerance is an important factor to consider when studying resistance evolution, since it potentially elevates the probability of *de novo* resistance mutations to arise during treatment by providing a reservoir of viable cells from which resistant mutants can emerge. In addition, it might support the selection of the acquired resistance mutations as the combined effect of tolerance and resistance might reduce the chance that a partially resistant mutant will be lost during treatment with high antimicrobial concentrations (61). Consequently, high levels of tolerance might open additional evolutionary trajectories by favoring low-impact mutations that would otherwise not provide sufficient protection for enrichment (31).

In our previous work (16), tolerance was only assessed for one specific combination of *Raoultella* and *Pseudomonas* strains (isolated from sample 3.8). However, since isolates belonging to the genera *Pseudomonas* or *Raoultella* were frequently encountered together in the sampled brewery biofilms, we first explored whether an increased tolerance of *Pseudomonas* in the presence of *Raoultella* could also be observed in isolates obtained from three other brewery samples. As before, we hereto compared the tolerance of mono- and duo-species biofilms against preventive treatment with sulfathiazole (16). Tolerance to sulfathiazole is defined as the ratio between the number of biofilm cells in the presence and absence of treatment. Although the evaluated biofilms exhibited considerable variation in their absolute growth as well as in their final population composition, the interactions in the majority (4/5) of duo-species communities were competitive in nature. However, only in the community already explored in our previous work (isolates from sample 3.8) this competition was associated with an enhanced tolerance of *Pseudomonas* (Fig. S2). In contrast, *Raoultella* sp. did not exhibit increased tolerance in any of the duo-species biofilms.

**Fig 1:**
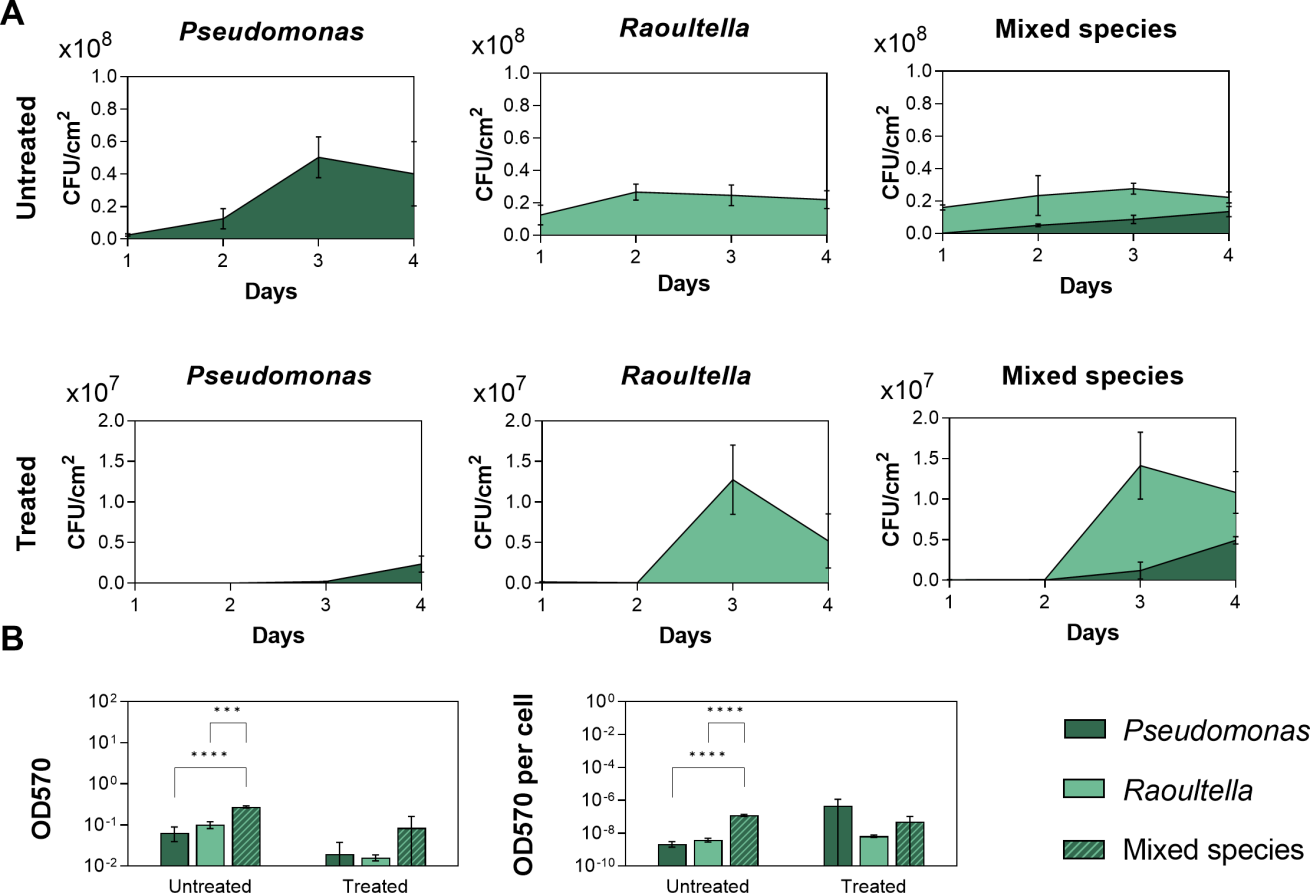
(**A**) Growth dynamics of mono- and duo-species biofilms in the absence or presence of sulfathiazole. The average of three biological repeats and standard deviation are shown. (**B**) EPS production in mono- and duo-species biofilm communities. Total and per cell EPS production in mono- and duo-species biofilms under untreated or treated conditions was quantified using a crystal violet assay. The average and standard deviation of three biological repeats are shown. An ordinary analysis of variance (ANOVA) with Tukey’s range test for multiple comparisons correction was used to compare the arithmetic means between the different samples (***P ≤ .001, ****P ≤ .0001).

In the following sections, we, therefore, focused on the interactions between the *Raoultella* and *Pseudomonas* strains isolated from sample 3.8 to investigate resistance evolution. Although this interaction was already briefly explored before (16), the identity of the specific strains was unknown, and the dynamics of the interaction were not characterized. Hence, we first performed whole genome sequencing and identified these strains as *Raoultella terrigena* and *Pseudomonas rhodesiae*. We then followed the population dynamics over time and found that, in monospecies-untreated conditions, *R. terrigena* rapidly reached its maximum population size, while the cell number of *P. rhodesiae* gradually increased over the first 3 days (Fig. 1A). The growth in untreated duo-species biofilms showed a similar trend with *Pseudomonas* progressively becoming the dominant species in the community. Treating monospecies communities with sulfathiazole resulted in a 1-day and 2-day growth delay of *R. terrigena* and *P. rhodesiae*, respectively, and a significant drop in their final population sizes. The duo-species conditions had a negligible effect on the dynamics and susceptibility of *Raoultella*. However, in the presence of *Raoultella*, the growth of *P. rhodesiae* started a day earlier than in monospecies-treated conditions. Possibly, these altered growth dynamics contribute to the enhanced tolerance of *Pseudomonas* in duo-species conditions observed after 4 days of treatment. The enhanced tolerance in duo-species conditions was not solely due to the inhibition of the competing *Raoultella* strain (competitive release) (16) as the cell number of *Pseudomonas* in duo-species treated conditions exceeded the treated monoculture level. These results thus indicate that the tolerance phenotype is induced in the duo-species biofilm. One of the key phenotypes determining the tolerance of biofilm bacteria is the production of EPS that form a protective layer and shield bacteria from antimicrobials (62, 63). Therefore, we quantified the EPS production in mono- and duo-species conditions via crystal violet staining (Fig. 1B). Both in treated and untreated conditions, the EPS production was increased in the duo-species community, albeit non-significantly in the presence of sulfathiazole. However, the EPS production per cell was not increased under treated conditions (Fig. 1B), indicating that the enhanced EPS production was due to the larger number of cells surviving the treatment in the duo-species community and that EPS is likely not the (sole) mechanism underlying the increased tolerance of *P. rhodesiae* in duo-species conditions.

### *Pseudomonas* evolves decreased susceptibility toward sulfathiazole solely in duo-species conditions

In order to study how the interspecies interactions influence the adaptation toward a long-term sulfathiazole treatment, we evolved both mono- and duo-species biofilms for a period of 80 days. In addition to the sulfathiazole-treated biofilms, an untreated control was included. Each condition consisted of 20 biological repeats, resulting in 120 different lineages. Serial transfer of biofilm cells was performed every 4 days, while biofilm and planktonic cells were quantified every 8 days (starting on day 4 and ending on day 76). A 4-day cycle was chosen as *P. rhodesiae* seems to only start noticeably growing during the fourth day of exposure to sulfathiazole (Fig. 1A).

**Fig 2:**
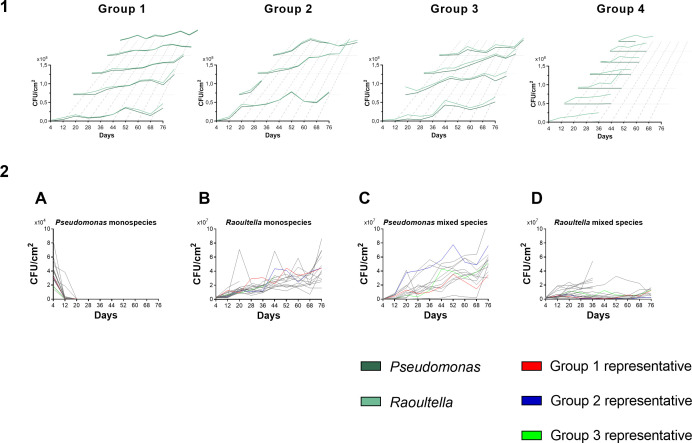
(1) Population size progression (CFU/cm^2^) of biofilm communities over the course of the evolution experiment (76 days) in the presence of sulfathiazole (categorized repeats). Populations of constituent species are represented with dark green for *Pseudomonas* sp. and light green for *Raoultella* sp. Individual lines show different biological repeats, with repeats corresponding to the same type of population dynamics scenario (i.e., group 1, 2, 3, or 4) congregated in the same plot. The repeats representing group 1, 2, or 3 are positioned frontally (2). Population size progression (CFU/cm^2^) of biofilm communities over the course of the evolution experiment (76 days) in the presence of sulfathiazole (merged repeats). (**A**) *P. rhodesiae* monospecies biofilms. (**B**) *R. terrigena* monospecies biofilms. (**C**) *P. rhodesiae* subpopulation of duo-species biofilms. (**D**) *R. terrigena* subpopulation of duo-species biofilms. Individual lines (20) show different biological repeats, with repeats representing group 1, 2, or 3 highlighted in red, blue, and green, respectively. Cross-contamination with other bacterial species was detected in monospecies communities in one of the repeats from group 2, which resulted in premature termination of the evolution experiment for this repeat. The evolution of duo-species communities was stopped when it was not possible to detect one of the constituent species anymore.

The cell counts of bacterial communities subjected to sulfathiazole treatment throughout the evolution experiment are shown in Fig. 2 (the focal repeats for later analyses, see below for details on categorization of repeats and category representatives) and Fig. S3. Invariably, all monospecies *P. rhodesiae* lineages exposed to sulfathiazole went rapidly extinct, whereas in duo-species conditions, the biofilm productivity of *P. rhodesiae* progressively increased throughout the evolution experiment for the majority (13/20) of the repeats (Fig. 2). The productivity of *Raoultella* during treatment gradually increased in monospecies conditions, while it generally remained constant if *Pseudomonas* was present (Fig. 2). Consequently, *Pseudomonas* progressively became the predominant species of the treated duo-species community. More specifically, three different trends in population dynamics were observed in the treated duo-species biofilms. In the first scenario, *Pseudomonas* gradually increased its population size throughout the evolution experiment (group 1: five repeats). In the second scenario, *Pseudomonas* showed a strong and rapid increase in population size (group 2: four repeats). Lastly, in some repeats, the gradual increase of the population of *Pseudomonas* was also associated with enhanced productivity of *Raoultella* (group 3: four repeats). The remaining repeats in which *Pseudomonas* did not evolve resistance were assigned to group 4. Importantly, both mono- and duo-species biofilm productivities did not increase greatly in the absence of sulfathiazole (Fig. S4), indicating that the enhanced biofilm productivity of *Pseudomonas* in the treated duo-species biofilms is due to adaptation toward the sulfathiazole treatment. The composition of untreated duo-species biofilms remained fairly constant over the course of the evolution experiment (Fig. S4C and D).

Aside from monitoring the growth of the biofilms, the population size of the planktonic cells above the biofilm was also quantified over time via optical density measurements (Fig. S5). These measurements did not allow us to differentiate between the two species in duo-species conditions and only showed the total productivity of both species combined. Similar to the biofilm cells, most repeats of *R. terrigena* showed a gradual increase in growth in the treated monospecies conditions, whereas all lineages of *P. rhodesiae* went extinct. However, the minor increase in treated duo-species productivity indicates that the strong increase of *Pseudomonas* in treated duo-species conditions is biofilm specific. In the absence of treatment, the community productivity remained largely unchanged throughout the evolution experiment.

Although the enhanced initial tolerance of *P. rhodesiae* biofilm cells in duo-species conditions is likely not due to increased EPS production, we explored further whether the heightened survival of *Pseudomonas* during evolution in treated duo-species biofilms is correlated with an increase in EPS production (Fig. S6). The EPS production was found to vary greatly between different lineages and different time points, yet there appeared to be a gradual trend of increasing EPS levels over time in treated duo-species conditions (Fig. S6A). However, this increase in EPS was related to the increased population size, since correcting for the changes in population size showed that the EPS production per cell decreased over time (Fig. S6B). These results thus suggest that selection for stronger EPS production likely cannot explain the increased survival of *Pseudomonas* in the treated duo-species biofilms.

### The evolved resistance mechanism works independently of *Raoultella*

**Fig 3:**
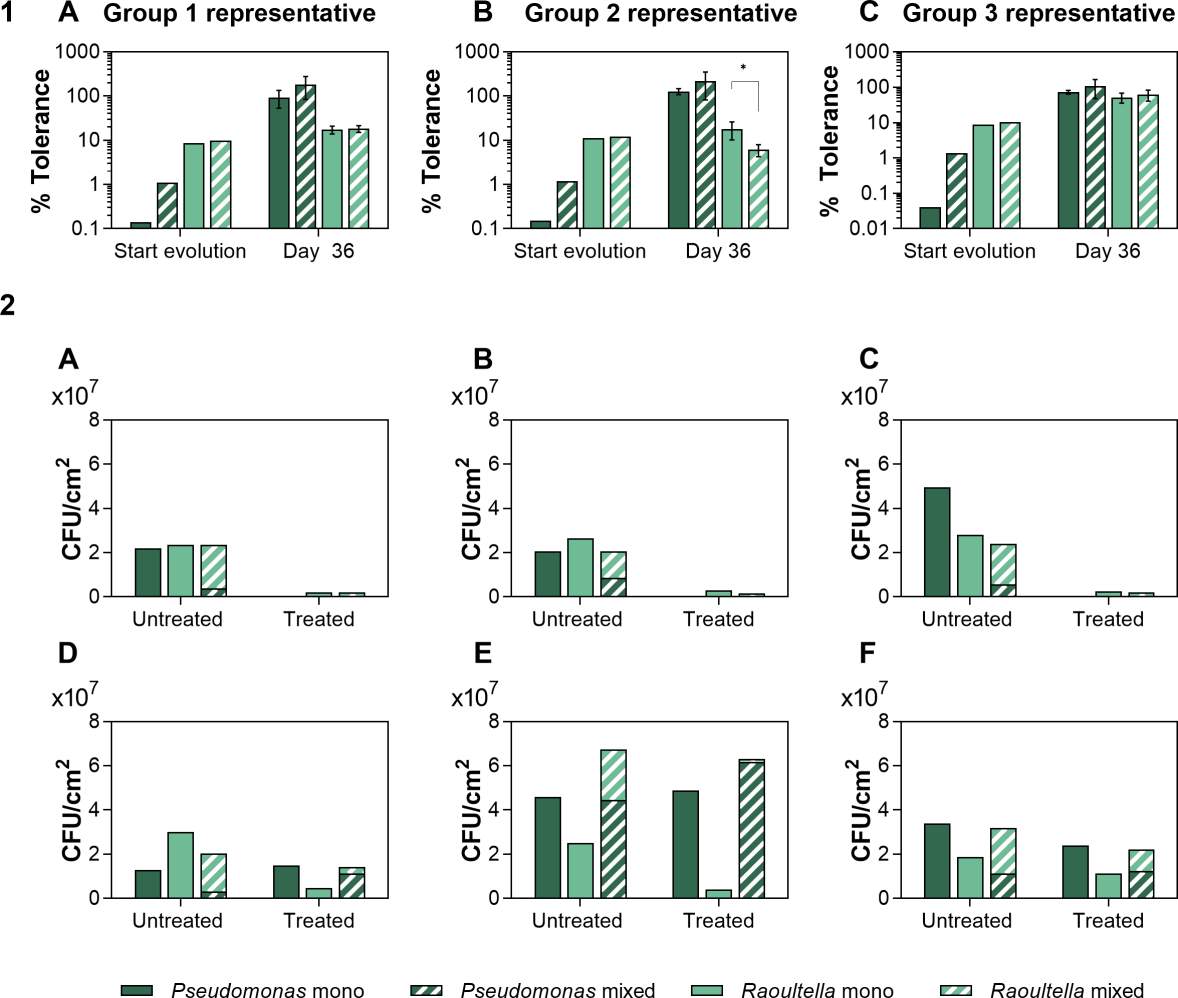
(1) Tolerance of *P. rhodesiae* and *R. terrigena* to sulfathiazole in mono- and duo-species biofilm communities at the start of the evolution experiment and on day 36. (**A-C**) The tolerance of a biofilm community originating from one distinctive colony isolated at the beginning of the evolution experiment is compared to the average of 10 biofilm communities originating from colonies of each species that were obtained from the treated duo-species biofilms on day 36 of the evolution experiment. The average and standard deviation of 10 biological repeats are shown. (**A**) Group 1 representative. (**B**) Group 2 representative. (**C**) Group 3 representative. To assess significance of differences between the observations of day 36, we employed a Brown-Forsythe and a Welch’s ANOVA test with Dunnett’s T3 correction for multiple comparisons (*P ≤ .05). (2) Growth of mono- and duo-species biofilm communities originating from isolated colonies of treated duo-species biofilms from day 0 or day 36 of the evolution experiment in the presence or absence of sulfathiazole. (**A–C**) Biofilm productivity of mono- and duo-species biofilm communities isolated at the beginning of the evolution experiment from representative of group 1 (**A**), representative of group 2 (**B**), or representative of group 3 (**C**). (D–F) Biofilm productivity of mono- and duo-species biofilm communities isolated on the day 36 of the evolution experiment from representative of group 1 (**D**), representative of group 2 (**E**), or representative of group 3 (**F**). Stacked columns represent the population structure of a mixed species community. The data are based on only one biological repeat, and hence, no robust statistical analyses were performed.

In order to study whether the decreased susceptibility to sulfathiazole of the *P. rhodesiae* lineages evolved in treated duo-culture is due to a further increase in mixed-species tolerance or whether a resistance mechanism independent of *R. terrigena* has developed, we isolated *P. rhodesiae* and *R. terrigena* cells after 36 days of evolution in the treated duo-species conditions. Specifically, we selected 10 colonies of each species from three different repeats, each representing one of the distinctive scenarios in the evolutionary dynamics of the treated duo-species communities (group 1, 2, or 3; *Pseudomonas* went extinct in group 4). The selected colonies were then used to inoculate mono- and duo-species biofilms in the presence and absence of sulfathiazole. Overall, colonies obtained from the same population behaved similarly (Fig. S7-1), suggesting that selective sweeps of beneficial mutations occurred in these bacterial communities (64). Subsequently, we compared the sulfathiazole susceptibility of these strains evolved in the treated duo-species biofilms and the ancestors in both mono- and duo-species conditions (Fig. 3). Remarkably, the evolved *Pseudomonas* strains were also no longer susceptible to sulfathiazole treatment in monospecies conditions. These findings thus indicate that, although adaptation toward sulfathiazole treatment required the presence of *Raoultella*, it resulted in the development of a resistance mechanism that works independently of *Raoultella*. As this resistance mechanism already provides complete protection against the treatment in monospecies conditions, interspecies interactions can no longer further decrease susceptibility to sulfathiazole. However, in the repeat representing group 1, the presence of *Raoultella* increased the tolerance of *Pseudomonas* to a level above 100%. This phenomenon could be attributed to the inhibition of *Raoultella* in treated duo-species conditions, resulting in a competitive release of the *Pseudomonas* strain (Fig. 3-1B). A similar trend occurred in the representative of group 2; however, it did not result in a significantly increased tolerance. The repeat representing group 3 showed the lowest level of competitive release, likely due to the low susceptibility to sulfathiazole of the evolved *Raoultella* strain in this repeat. Furthermore, sulfathiazole also did not inhibit the planktonic growth of the evolved *Pseudomonas* strains (Fig. S7-2), indicating the evolved resistance mechanism is not biofilm specific. In the absence of treatment, there does not seem to be a considerable evolution of resistance toward sulfathiazole (Fig. S8Fig. S8).

**Fig 4:**
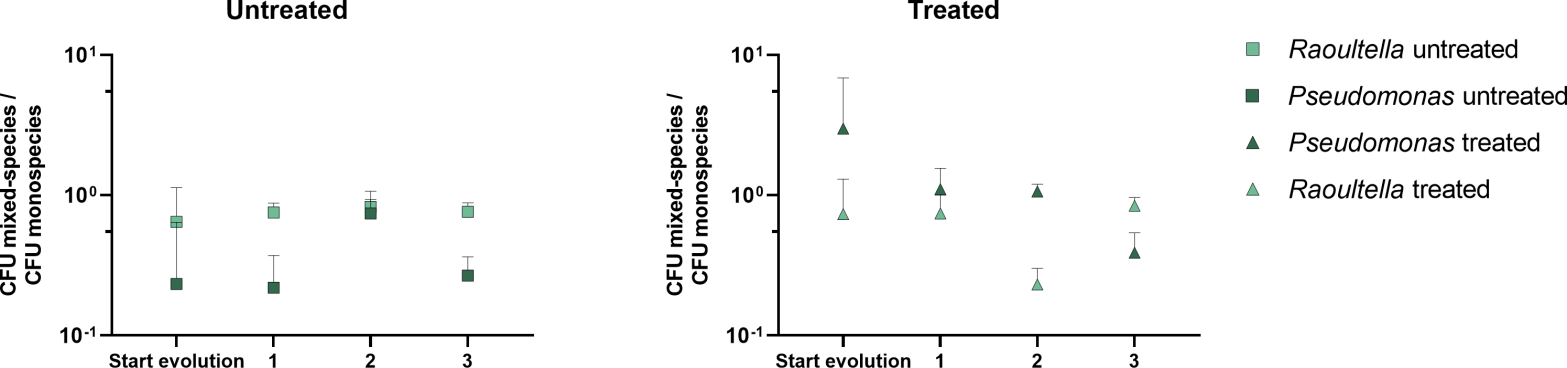
Evolution of interspecies interactions in the presence or absence of sulfathiazole. The ratios between the growth of duo- and monospecies biofilms of repeats representing groups 1, 2, and 3 at the beginning and on day 36 of the evolution experiment are shown. Cooperative interactions are defined to occur when both species experience an increased growth in the duo-species conditions (>1). Commensal interactions are defined to occur when one species experiences an increased growth (>1), while the other species is neither harmed nor has any benefit (1). Competitive interactions are defined to occur when at least one species is harmed (<1). A Brown-Forsythe and a Welch’s ANOVA test with Dunnett’s T3 correction for multiple comparisons were used to compare the arithmetic means between the different samples.

In addition, we studied how the prolonged co-evolution between *Pseudomonas* and *Raoultella* altered the interspecies interactions in different duo-species communities. We calculated the ratio between the mono- and duo-species productivity for every selected day 36 clone (Fig. 4) in order to assess to what extent the growth of a species is inhibited in duo-species conditions. This allowed quantifying the level of competition that takes place (47). As expected, based on prior results (16), interspecies competition initially prevailed in the absence of sulfathiazole. Similar results were observed under treatment, with interactions between *R. terrigena* and *P. rhodesiae* ranging from exploitative to competitive. Overall, the interactions showed a similar trend after 36 days of evolution, indicating that there was no evolution toward the relaxation of competition. This preservation of competition further supports that competitive release could occur upon treatment. In addition, a lack of cooperative interactions in presence of sulfathiazole is consistent with the evolution of resistance mechanisms in *P. rhodesiae* independent of *R. terrigena*.

### Increased population size cannot explain the increased resistance evolution of *Pseudomonas* in duo-species conditions

**Fig 5:**
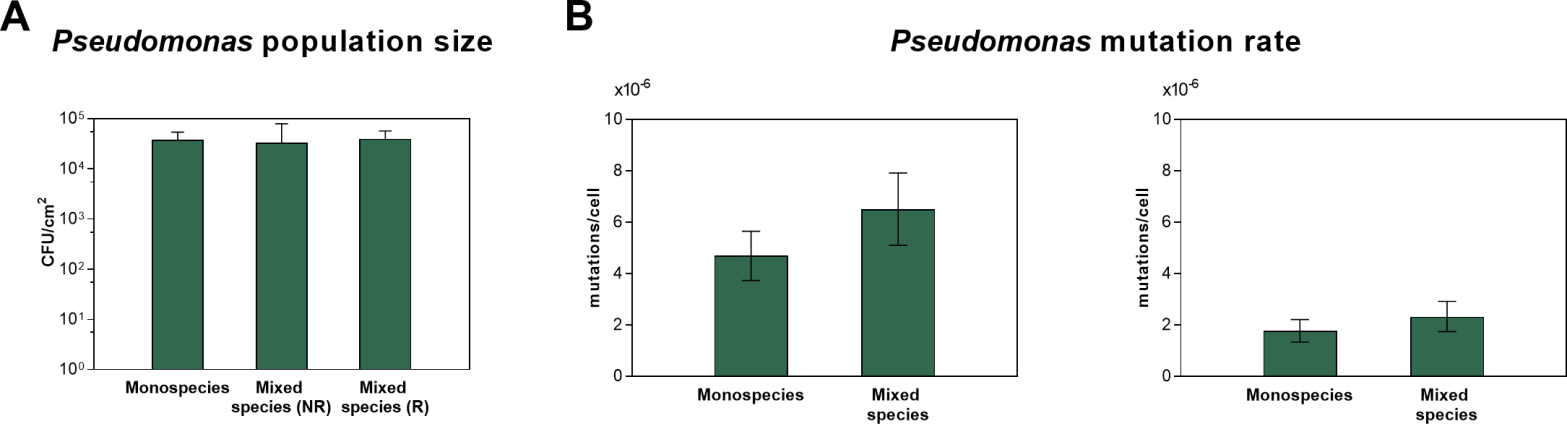
(**A**) The biofilm population size of *P. aeruginosa* in mono- and duo-species conditions on day 4 of the evolution experiment in the presence of sulfathiazole. The communities in duo-species conditions were grouped based on resistance development (R, resistance; NR, no resistance). The average and standard deviation of the biological repeats are shown. A Brown-Forsythe and a Welch’s ANOVA test with Dunnett’s T3 correction for multiple comparisons were used to compare the arithmetic means between the different samples. (**B**) The estimated mutation rates of *P. rhodesiae* within monospecies communities and duo-species biofilm communities for two independent repeats. The estimated mutation rates of *P. rhodesiae* are plotted with their corresponding 95% confidence intervals. Significant differences were evaluated using the likelihood ratio test.

The strongly enhanced survival rate and resistance evolution of *P. rhodesiae* in co-culture with *R. terrigena* suggest that the increased initial tolerance of *Pseudomonas* promotes resistance by increasing the pool of viable cells (i.e., population size) from which resistant mutants can emerge or by supporting the selection of partially resistant mutants (61). To distinguish between both mechanisms, we compared the different parallel lineages and assessed whether the population size of *Pseudomonas* in the treated biofilm communities could be correlated to whether the evolution of resistance occurred in these communities. Note that increased tolerance does not directly imply increased absolute population size, since tolerance is defined as a relative measure comparing treated populations with untreated populations, which can differ in size themselves. We did not observe a significant difference in the size of *Pseudomonas* population at day 4 of the evolution between the lineages where *Pseudomonas* adapted to the treatment and the lineages where *Pseudomonas* went extinct (Fig. 5A). Thus, if increased tolerance underlays the development of resistance in duo-species conditions, it is more probable due to tolerance enhancing the selection of partially resistant mutants and opening up novel evolutionary trajectories rather than by increasing the pool of viable cells.

### Increased mutation rate does not explain the increased resistance evolution in duo-species conditions

In addition to tolerance, interspecies interactions can also influence other factors that contribute to resistance development, an example being the mutation rate. It was previously reported that stress induced by competitive interactions within biofilm communities can enhance the mutation rate by activating the low-fidelity DNA polymerases (29, 65). The activation of these low-fidelity DNA polymerases is thought to serve as an adaptive response in stressed cells in order to produce descendants that are better adapted to the present stressors (66, 67). To evaluate whether the mutation rate contributed to the increased resistance evolution in duo-culture, we compared the mutation rate of *P. rhodesiae* in mono- and duo-species biofilms (Fig. 5B). Hereto, a fluctuation test was modified for the use with biofilm cultures (see Materials and Methods). Mutation rates were slightly increased in the duo-species biofilm; however, no statistically significant changes were observed. Differences in mutation rate, therefore, do not likely significantly contribute to the enhanced resistance evolution in duo-species conditions (Fig. 5B).

### Horizontal gene transfer does not contribute to the increased resistance evolution in duo-species conditions

A well-described mechanism specific to duo-species communities is the exchange of resistance-associated genes between species via horizontal gene transfer. In order to identify the acquisition of genetic material by *P. rhodesiae* in duo-species conditions, whole genome sequencing was performed. Hereto, the genomic material of the ancestors and evolved strains from treated and untreated duo-species communities at day 36 of repeats representing groups 1, 2, and 3 was collected. The evolved sequences were compared to the reference genome, and the data were screened for signs of horizontal gene transfer (including the existence of plasmids initially not present). Furthermore, the *de novo* assemblies of the reads of the evolved strains of *Pseudomonas* that did not align to the reference genome were evaluated for traces of horizontal gene transfer that were missed in the previous step. This bioinformatic analysesis did however not detect the exchange of genetic material in our samples that would explain the newly acquired resistance against sulfathiazole in *P*. *rhodesiae*.

### Resistance-associated genes are mutated after long-term treatment with sulfathiazole

The coevolution between *P. rhodesiae* and *R. terrigena* resulted in *Pseudomonas* becoming resistant to sulfathiazole in a way that is independent of the presence of *Raoultella*. As suggested by the analyses above, this resistance is likely not a direct effect of the population size, increased mutation rate, or horizontal gene transfer (HGT). Therefore, we hypothesized that the increased tolerance during the initial stages of coevolution allows exploring evolutionary trajectories, consisting of low-frequency mutations that would otherwise not be selected.

Comparing the variants observed in the evolved repeats with their matching ancestral strains allows identifying mutations that were selected under coexistence in the presence and absence of antimicrobial selection. We selected—as potentially adaptive—those variants that increased more than 10% in allele frequency in the evolved clonal pools versus the ancestral genome (Table S1). Surprisingly, few variants were present at high allele frequency in clonal pools isolated from populations cultivated in the presence of sulfathiazole. We did not find a single mutation that was present in all three parallel populations subjected to sulfathiazole and absent in the populations grown in the absence of the antimicrobial. In addition, we did not identify any high-frequency mutation in known resistance-associated genes. All treated populations did show mutations in multidrug-efflux-pump-coding genes, such as *htpx*, *gacA*, and *macB*, at low frequencies. Mutations in these genes have previously been linked to antimicrobial resistance in *Pseudomonas* (68–72). However, these genes were also mutated in strains evolved in the absence of sulfathiazole, indicating that such mutations can also occur as an adaptation to the growth conditions and thus are not necessarily linked to the resistance phenotype.

## DISCUSSION

The evolution of resistance toward antimicrobials has been extensively studied and characterized (64, 73–76). However, the vast majority of this research investigated resistance development in well-mixed and monospecies environments (1–4). These conditions disagree with how bacteria typically live: embedded in structured and diverse communities (16, 24, 25, 77–79). While it has been reported that these mixed-species consortia are often more tolerant than their monospecies counterparts (14, 16, 24, 80–83), studies on resistance development in structured mixed-species communities are scarce. Two studies compared the resistance development of mono- and duo-species biofilm communities to daily antimicrobial treatment but observed no resistance development in either condition (84, 85). While repeated treatment of a mixed-species biofilm consisting of the resident microflora in a cheese factory showed gradual resistance development, the evolution of the same species in monoculture was not investigated (11). The effect of social interactions on resistance development in mixed-species biofilms thus remains unclear.

Therefore, we characterized how a *Pseudomonas* isolate, obtained from a biofilm contamination in a brewery, adapts to a continuous sulfathiazole treatment in a monoculture and in the presence of a co-isolated *Raoultella* strain. We had previously reported that the interactions among these two species are characterized by competition and had resulted in an enhanced tolerance of *Pseudomonas* toward sulfathiazole (16). Here, we serially transferred the *Pseudomonas* and *Raoultella* strains for 80 days in mono- and co-culture and showed that the social interactions in the mixed-species biofilm also strongly influence resistance development. Indeed, *Pseudomonas* commonly became less susceptible toward treatment with sulfathiazole in the presence of a co-isolated *Raoultella* strain, while no resistance development and extinction were observed in monospecies conditions. Moreover, the strains evolved in duo-species conditions retained their decreased susceptibility to treatment in monospecies conditions, indicating that the evolved resistance mechanism works independently of *Raoultella*. Remarkably, looking at the evolution of social interactions themselves, the evolved *Pseudomonas* and *Raoultella* strains retained similar levels of competition as the ancestors. This is in contrast with the limited number of previous studies on the long-term evolution of interactions in mixed-species communities. These studies in general observed an increase in community productivity and a decrease in competition (86–88).

An increase in the initial population size of *Pseudomonas* due to enhanced tolerance is unlikely to fully explain the large discrepancy in successful adaptation between mono- and duo-species conditions during treatment, since the distribution of *Pseudomonas* population sizes after 4 days of evolution was statistically not different for the lineages that adapted to treatment compared to the lineages that went extinct. Next to population size, the mutation rate can influence the supply of emerging resistant mutants (89). It was previously reported that co-culture can increase mutation rate (29, 65), possibly due to bacterial competition resulting in enhanced stress (83, 90, 91), a known inducer of mutation rate (66, 92, 93). However, the mutation rate was also not increased in our duo-species biofilms. Alternatively, bacteria in mixed-species communities could acquire novel resistance mechanisms via horizontal gene transfer, especially in biofilms where the high-cell-density and structured environment leads to higher conjugation rates. Moreover, biofilm matrix typically contains a high amount of extracellular DNA, enhancing the potential for transformation (10, 21). However, whole genome sequencing of resistant colonies showed no exchange of genetic material between the two species.

Overall, these data thus indicate that the enhanced resistance development in the presence of *Raoultella* is not due to an increased supply of mutants. Instead, social interactions more likely influence the selection of resistant phenotypes and open up new evolutionary trajectories. Possibly, the strong inhibition experienced by *Pseudomonas* during treatment in monospecies conditions severely limits the occurrence of sufficiently resistant mutants (94), whereas the enhanced tolerance in duo-species conditions could expand the number of viable mutants, i.e., certain mutations might only provide sufficient resistance and enable growth in duo-species conditions. The genomic analysis clearly indicates that the acquisition of resistance toward sulfathiazole was a complex phenomenon and a phenotype for which no single optimal solution exists (no clear single selection sweep as is often observed for other antimicrobial selection regimes) (95). The analysis supports that resistance depends on trajectories that include mutations that only confer weak resistance and which eventually, when conditioned in a specific genetic background, might result in the sufficient resistance phenotype. It also points toward additional mechanisms that confer plasticity independent of genomic alterations (epigenetics).

As the use of natural isolates severely limits the ability to unravel the underlying mechanisms of tolerance and its impact on resistance due to the highly challenging nature of genetically engineering the natural isolates, we are currently performing additional mechanistic work employing model microbial species in order to further elucidate the link among social interactions, tolerance, and resistance development in mixed-species biofilms.

### Conclusion

Antimicrobial resistance is one of the most studied bacterial properties due to its enormous clinical and industrial relevance; however, most research focuses on resistance development of a single species in isolation. Here, we showed that resistance evolution of brewery isolates can differ greatly between mono- and mixed-species conditions. *Pseudomonas* readily developed resistance toward sulfathiazole if co-cultured in the presence of *Raoultella*, whereas all monospecies lineages rapidly went extinct. While evolution of sulfathiazole resistance required the presence of *Raoultella*, the acquired resistance mechanism is independent of *Raoultella* and also provides protection in monospecies conditions. Overall, the current study emphasizes the need of considering the social interactions in microbial communities when evaluating antimicrobial treatments and resistance evolution.

## Data Availability

The annotated ancestral genome of *P. rhodesiae* as well as the raw sequencing data of the pools of clones of the above-mentioned species evolved in the absence or presence of sulfathiazole under duo-species conditions are accessible in the NCBI data repository (BioProject: PRJNA972615 and SRA: SRR25440063, SRR25440064, SRR25440065, SRR25440066, SRR25440067, and SRR25440068).

## SUPPLEMENTAL MATERIAL

Figure S1 aem.01155-23-s0001.png Supplementary Figure 1.

Supplementary Figure Legends aem.01155-23-s0002.docx Supplementary Figure Legends.

Figure S2 aem.01155-23-s0003.tif Supplementary Figure 2.

Figure S3 aem.01155-23-s0004.tif Supplementary Figure 3.

Figure S4 aem.01155-23-s0005.tif Supplementary Figure 4.

Figure S5 aem.01155-23-s0006.tif Supplementary Figure 5.

Figure S6 aem.01155-23-s0007.tif Supplementary Figure 6.

Figure S7 aem.01155-23-s0008.tif Supplementary Figure 7.

Figure S8 aem.01155-23-s0009.tif Supplementary Figure 8.

Table S1 aem.01155-23-s0010.docx Supplementary Table 1.

ASM does not own the copyrights to Supplemental Material that may be linked to, or accessed through, an article. The authors have granted ASM a non-exclusive, world-wide license to publish the Supplemental Material files. Please contact the corresponding author directly for reuse.
