# Supplementary figures and images for: Competitive interactions facilitate resistance development against antimicrobials
Source: Appl Environ Microbiol. 2023 Oct 11;89(10):e01155-23. doi: 10.1128/aem.01155-23 (PMC10617502; doi:10.1128/aem.01155-23)

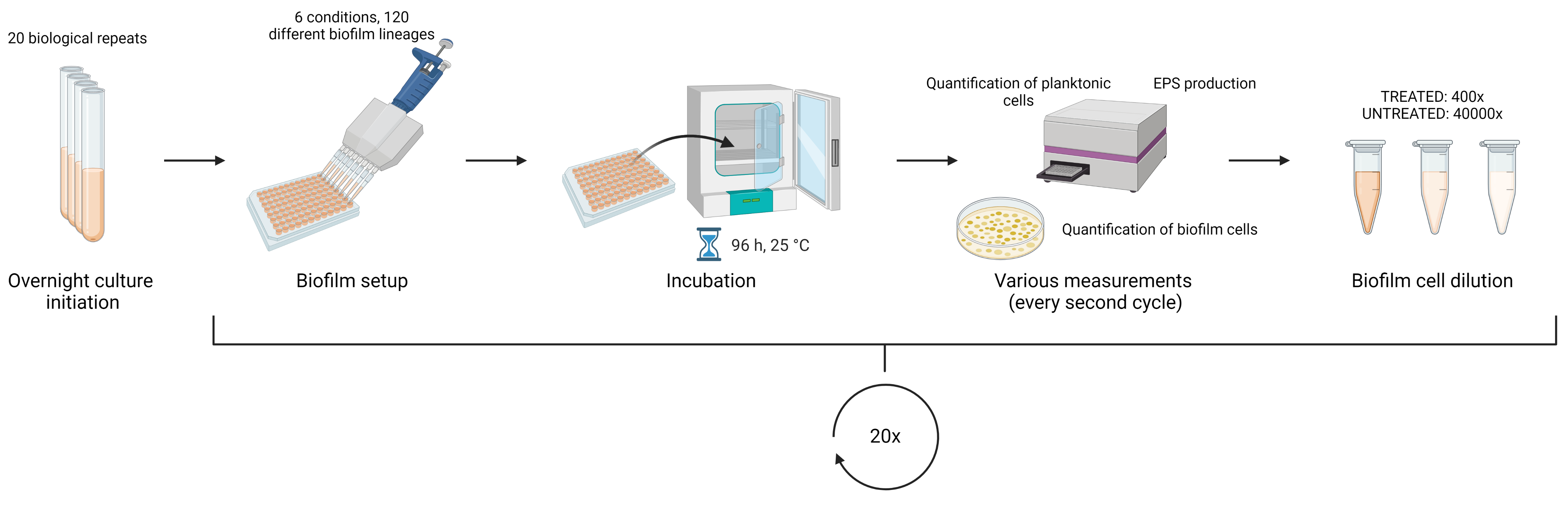

Supplement: Figure S1 — Supplementary Figure 1. [file aem.01155-23-s0001.png]

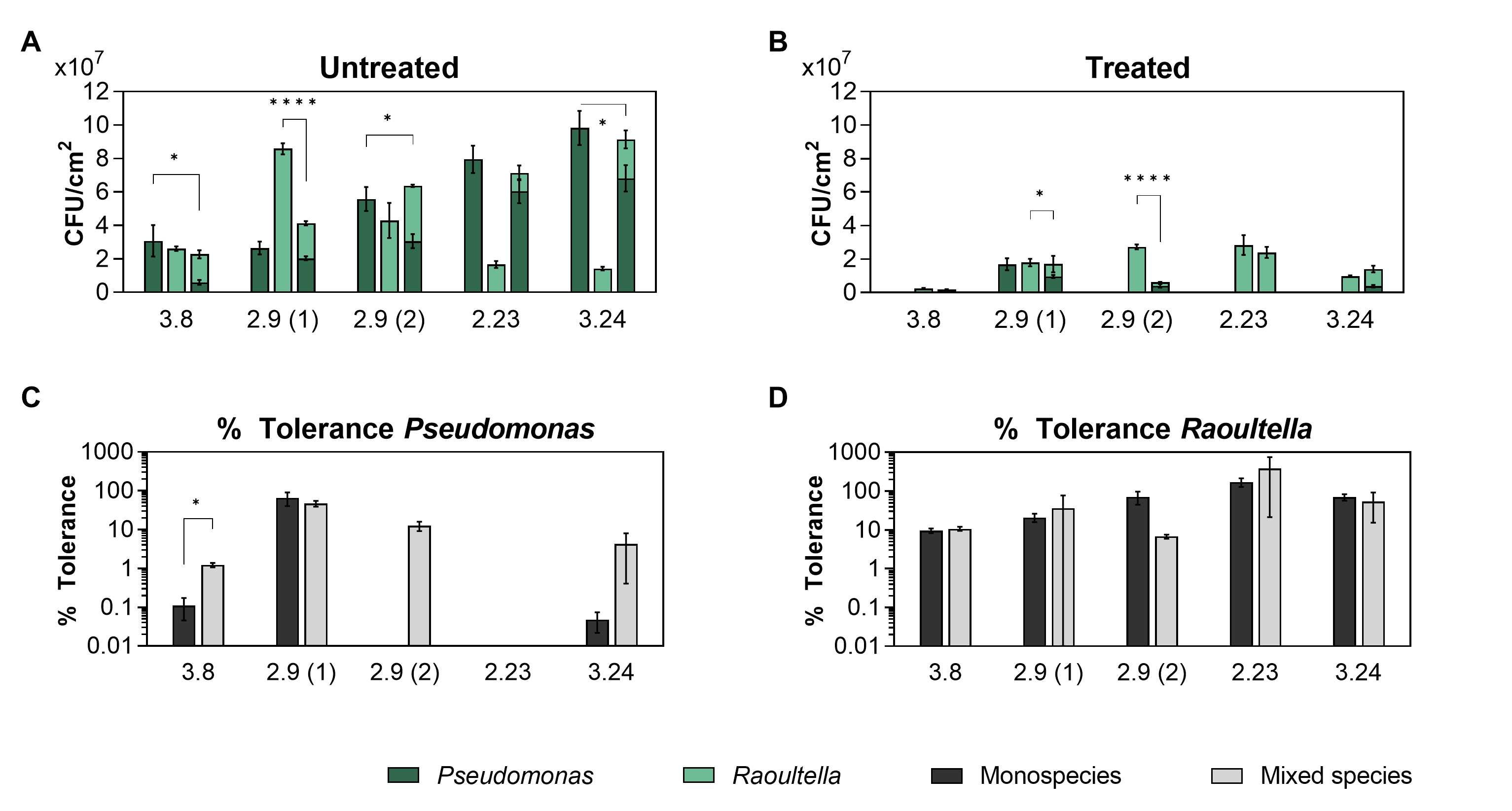

Supplement: Figure S2 — Supplementary Figure 2. [file aem.01155-23-s0003.tif]

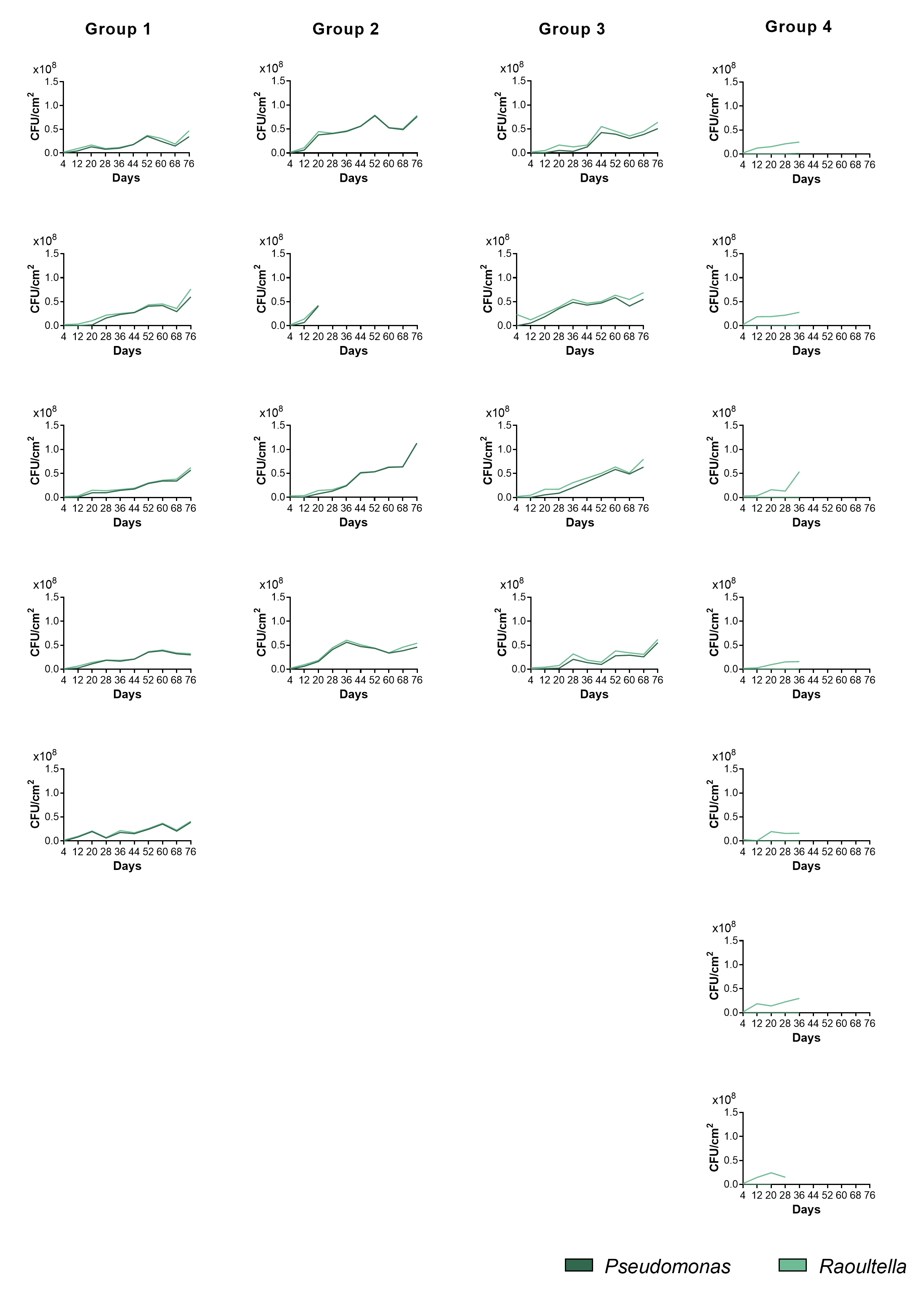

Supplement: Figure S3 — Supplementary Figure 3. [file aem.01155-23-s0004.tif]

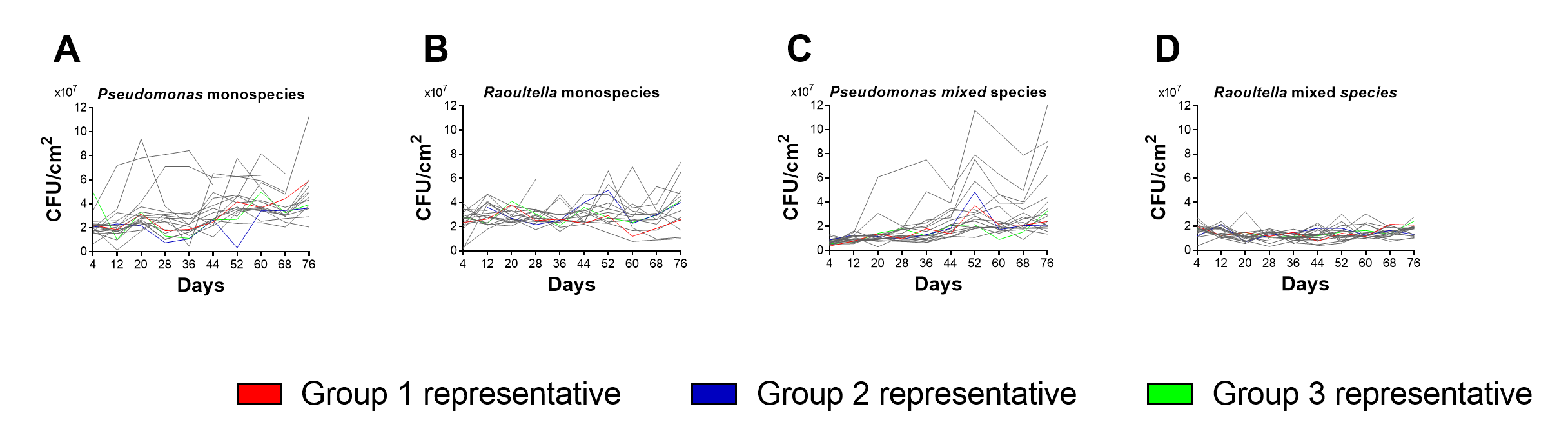

Supplement: Figure S4 — Supplementary Figure 4. [file aem.01155-23-s0005.tif]

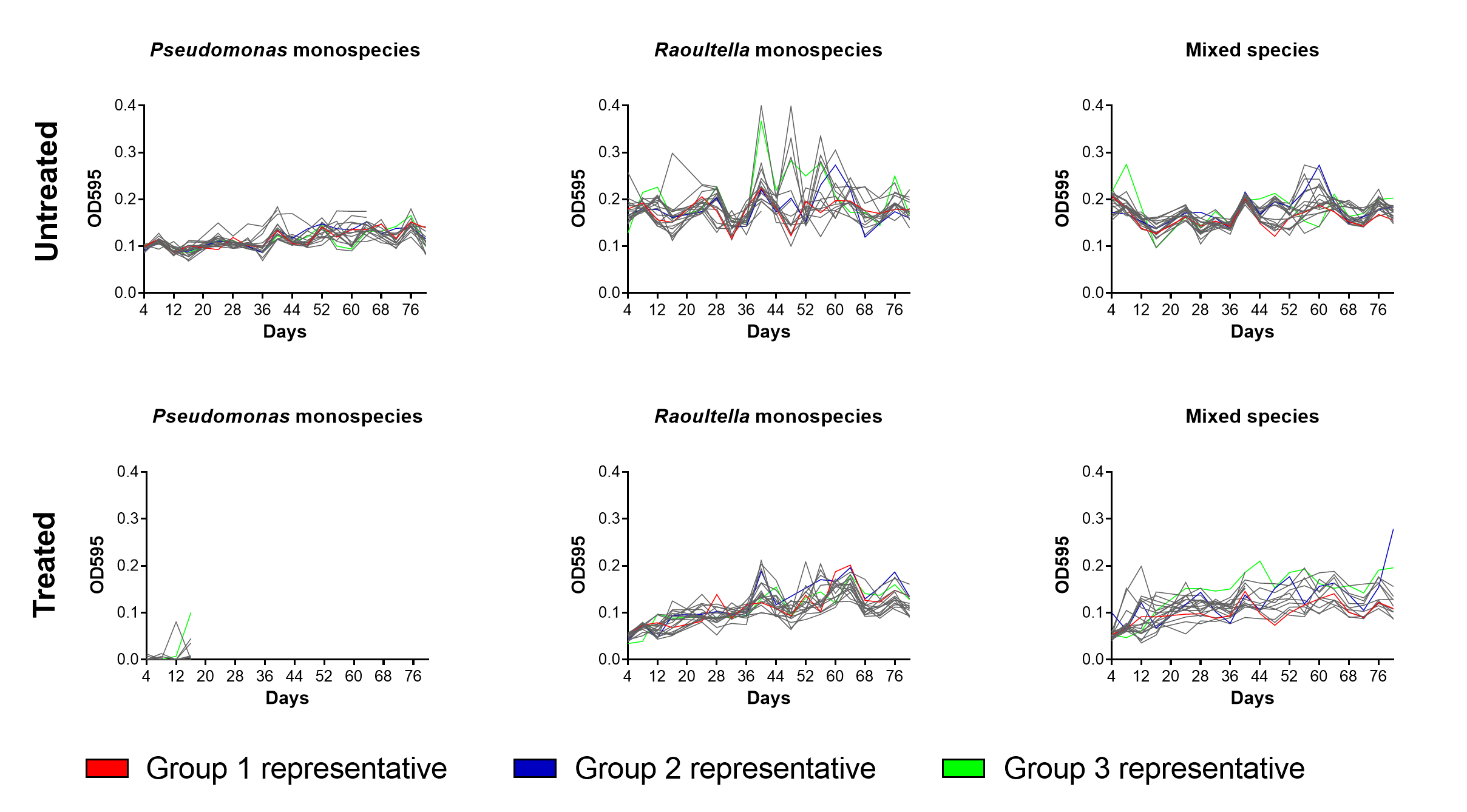

Supplement: Figure S5 — Supplementary Figure 5. [file aem.01155-23-s0006.tif]

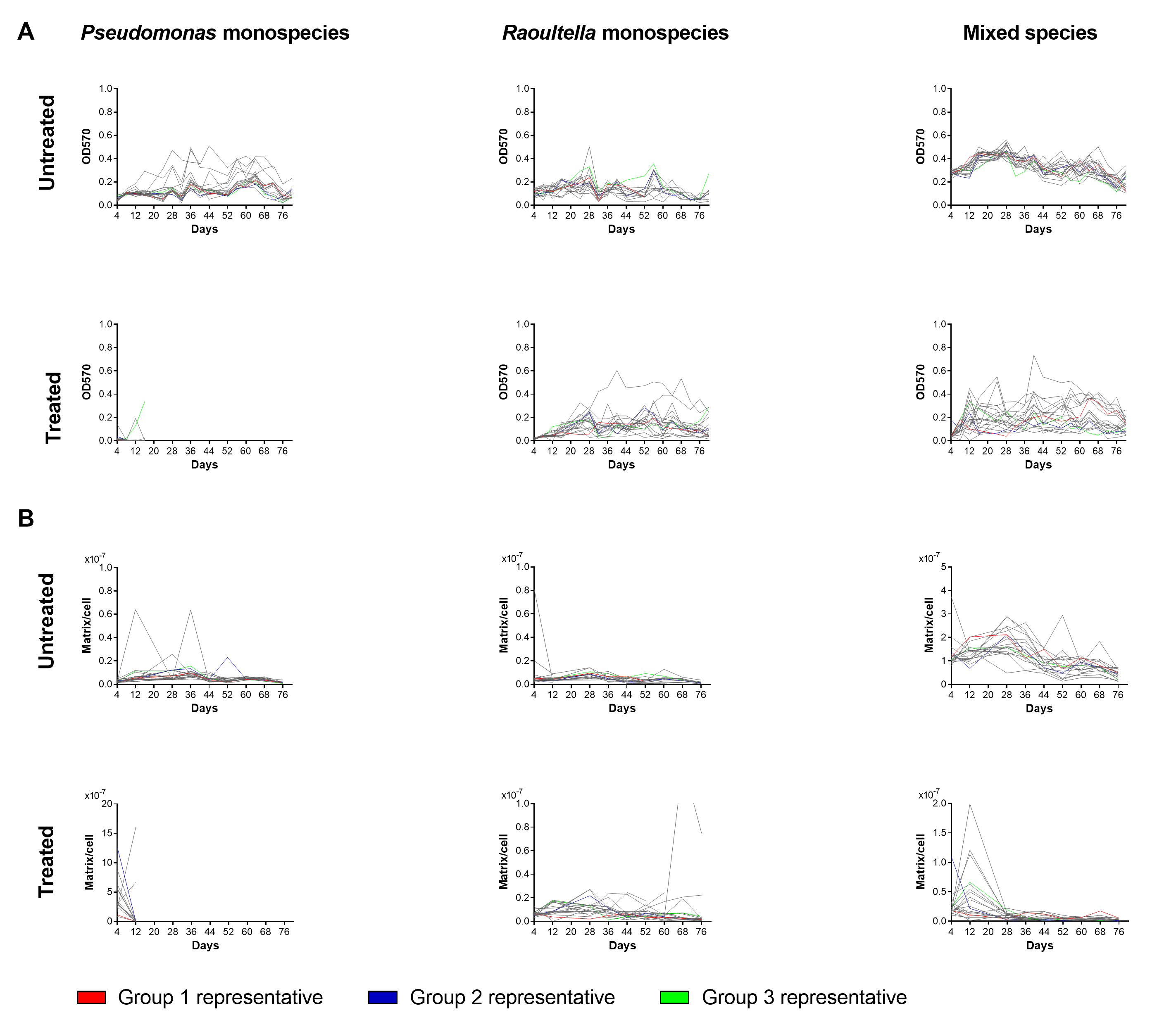

Supplement: Figure S6 — Supplementary Figure 6. [file aem.01155-23-s0007.tif]

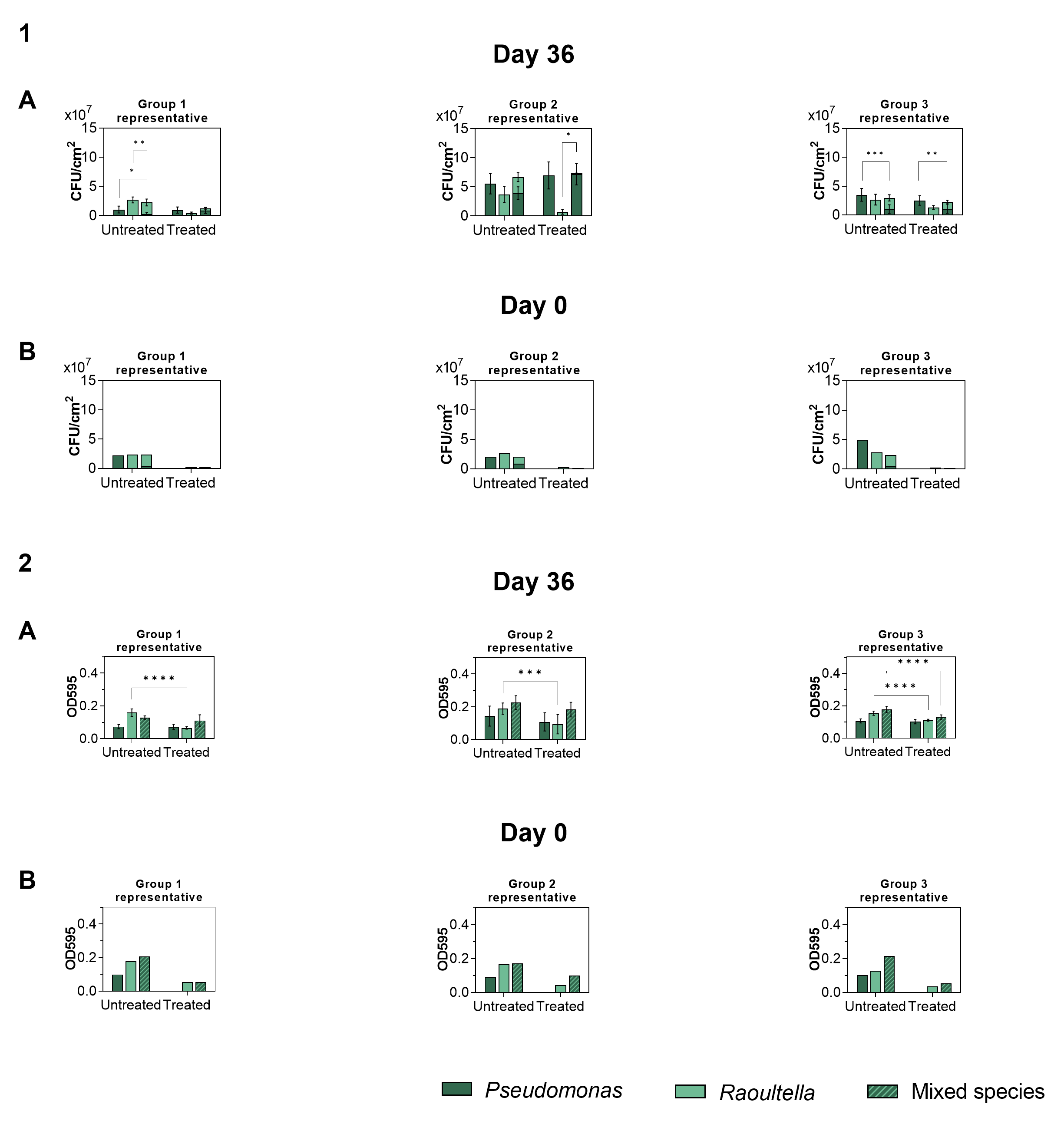

Supplement: Figure S7 — Supplementary Figure 7. [file aem.01155-23-s0008.tif]

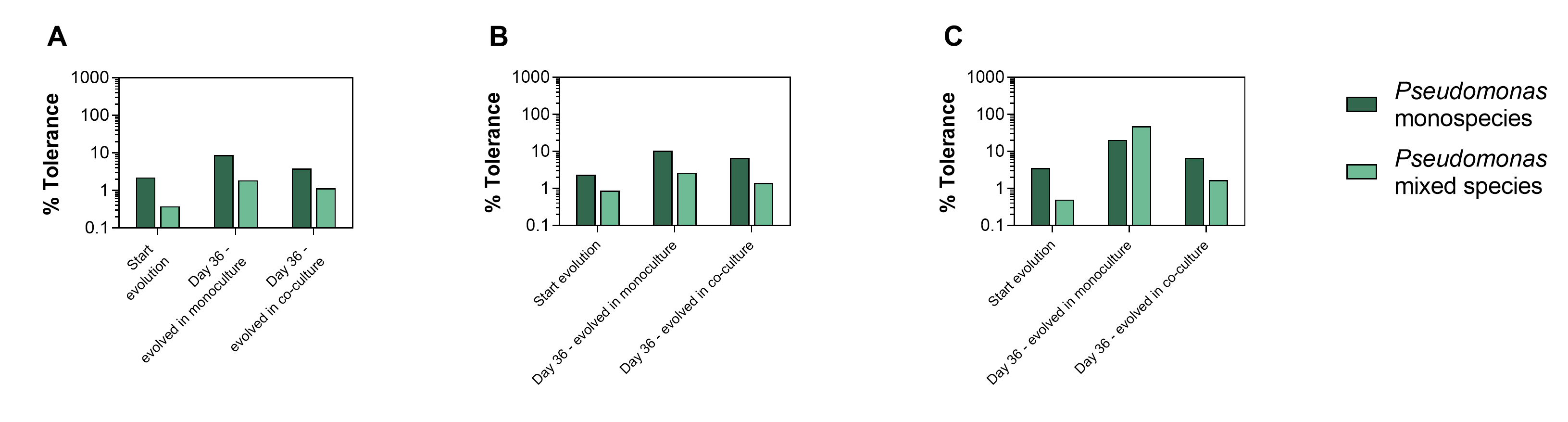

Supplement: Figure S8 — Supplementary Figure 8. [file aem.01155-23-s0009.tif]
