# Supplementary material for: Competitive interactions facilitate resistance development against antimicrobials
Source: Appl Environ Microbiol. 2023 Oct 11;89(10):e01155-23. doi: 10.1128/aem.01155-23 (PMC10617502; doi:10.1128/aem.01155-23)
Supplement: Supplementary Figure Legends [file aem.01155-23-s0002.docx]

# **SUPPLEMENTARY FIGURE LEGENDS**

**Figure S1: Schematic overview of the experimental evolution of mono- and duo-species biofilm communities.** Created with BioRender.com.

**Figure S2: Mono- and duo-species biofilm productivity of distinctive *Pseudomonas* sp. and *Raoultella* sp. isolates co-obtained from the same respective brewery sample.** A) Biofilm formation under untreated conditions. B) Biofilm formation under treated conditions (100 μM sulfathiazole). C) Tolerance of *Pseudomonas* sp. to sulfathiazole, expressed as percentage. D) Tolerance of *Raoultella* sp. to sulfathiazole, expressed as percentage. Two different strains of *Pseudomonas* sp. were isolated from sample 2.9, which were later combined with the same strain of *Raoultella* sp. to construct a duo-species biofilm. Stacked columns represent the population structure of a mixed species community. The average and standard deviation of three biological repeats are shown. In case of A, B and D, a Brown-Forsythe and a Welch’s ANOVA test with Dunnett’s T3 correction for multiple comparisons was used to compare the arithmetic mean of the productivity or tolerance of the focal species between mono- and duo-species conditions. For C we employed an ordinary ANOVA with Šidák correction for multiple comparisons (* = P≤0.05, **** = P≤0.0001).

**Figure S3: 1)** **Population size progression (CFU/cm^2^) of biofilm communities over the course of the evolution experiment (76 days) in the presence of sulfathiazole (all repeats, separate).** Populations of constituent species are represented with dark green for *Pseudomonas* sp. and light green for *Raoultella* sp. Repeats that correspond to the same type of population dynamics scenario (i.e., either group 1, 2, 3, or 4) congregated in the same column. The evolution of duo-species communities was stopped when it was not possible to detect one of the constituent species anymore. Cross-contamination with other bacterial species was detected in monospecies communities in one of the repeats from group 2, which resulted in premature termination of the evolution experiment for this repeat.

**Figure S4: Population size progression (CFU/cm^2^) of biofilm communities over the course of the evolution experiment (76 days) in the absence of sulfathiazole (all repeats, merged).** A) *P. rhodesiae* monospecies biofilms. B) *R. terrigena* monospecies biofilms. C) *P. rhodesiae* subpopulation of duo-species biofilms. D) *R. terrigena* subpopulation of duo-species biofilms. Individual lines (20) show different biological repeats, with repeats representing groups 1, 2, and 3 highlighted in red, blue, and green respectively. Cross-contamination with other bacterial species was detected in monospecies communities in one of the repeats from group 2, which resulted in premature termination of the evolution experiment for this repeat. The evolution of duo-species communities was stopped when it was not possible to detect one of the constituent species anymore.

**Figure S5: Population size progression (OD_595_) of planktonic cells over the course of the evolution experiment (76 days) in the absence and presence of sulfathiazole in mono- and duo-species communities.** Individual lines (20) show different biological repeats, with repeats representing groups 1, 2, and 3 highlighted in red, blue, and green respectively. Cross-contamination with other bacterial species was detected in monospecies communities in one of the repeats from group 2, which resulted in premature termination of the evolution experiment for this repeat. The evolution of duo-species communities was stopped when it was not possible to detect one of the constituent species anymore.

**Figure S6: A) Absolute EPS production (OD_570_) and B) EPS production per cell over the course of the evolution experiment (76 days) in the absence and presence of sulfathiazole in mono- and duo-species communities.** Individual lines (20) show different biological repeats, with repeats representing groups 1, 2, and 3 highlighted in red, blue, and green respectively. Cross-contamination with other bacterial species was detected in monospecies communities in one of the repeats from group 2, which resulted in premature termination of the evolution experiment for this repeat. The evolution of duo-species communities was stopped when it was not possible to detect one of the constituent species anymore.

**Figure S7: 1) Growth of mono- and duo-species biofilm communities in the presence or absence of sulfathiazole originating from isolated colonies of treated duo-species biofilms from day 0 or day 36 of the evolution experiment.** A) The productivity of mono- and duo-species biofilm communities at day 36 of the evolution experiment. Stacked columns represent the population structure of a mixed species community. The graphs portray the average and standard deviation of the productivity of ten biological repeats, each initiated from a distinctive colony from repeats representing groups 1, 2, and 3. A Brown-Forsythe and a Welch’s ANOVA test with Dunnett’s T3 correction for multiple comparisons were used to compare the arithmetic means between the different samples. B) One biofilm community of each species originating from a distinctive colony isolated from repeats representing groups 1, 2, and 3 at the beginning of the evolution experiment. The data are based on only one biological repeat and hence no robust statistical analyses were performed. **2) Growth of planktonic cells (OD_595_) in mono- and duo-species communities, originating from isolated colonies of treated duo-species biofilms from day 0 or day 36 of the evolution experiment,** **in the presence or absence of sulfathiazole.** A) The growth of planktonic cells measured by quantifying the optical density at 595 nm in mono- and duo-species communities at day 36 of the evolution experiment. The graphs portray the average and standard deviation of the growth of ten biological repeats, each initiated from a distinctive colony from repeats representing groups 1, 2, and 3. In case of group 1 representative, a Brown-Forsythe and a Welch’s ANOVA test with Dunnett’s T3 correction for multiple comparisons were used to compare the arithmetic mean of the growth of the focal or mixed-species between treated and untreated conditions. For repeats representing groups 2 and 3 we employed an ordinary ANOVA with Šidák correction for multiple comparisons (*** = P≤0.001, **** = P≤0.0001). B) One community of each species originating from a distinctive colony isolated from repeats representing groups 1, 2, and 3 at the beginning of the evolution experiment. The data are based on only one biological repeat and hence no robust statistical analyses were performed.

**Figure S8: Tolerance of *P. rhodesiae* to sulfathiazole in mono- and duo-species biofilm communities at the beginning of the evolution experiment and after 36 days of evolution in the absence of sulfathiazole.** A biofilm community originating from one distinctive colony isolated at the beginning of the evolution experiment is compared to a biofilm community originating from colonies that were obtained from the untreated mono- and duo-species biofilms on day 36 of the evolution experiment. Data portray only one biological repeat and hence no robust statistical analyses were performed. A) Group 1 representative. B) Group 2 representative. C) Group 3 representative.
