# Supplementary material for: Competitive interactions facilitate resistance development against antimicrobials
Source: Appl Environ Microbiol. 2023 Oct 11;89(10):e01155-23. doi: 10.1128/aem.01155-23 (PMC10617502; doi:10.1128/aem.01155-23)
Supplement: Table S1 — Supplementary Table 1. [file aem.01155-23-s0010.docx]

**Table S1: Modifications in the genetic code of *P. rhodesiae* evolved in the presence or absence of sulfathiazole and their corresponding protein effects and frequencies.** The column *Sample* designates the origin of the genetic material, specifically repeats representing groups 1, 2, and 3, and whether those *Pseudomonas* isolates were evolved in the presence (*treated*) or absence (*untreated*) of sulfathiazole. All the genetic material of the evolved isolates originates from day 36 of the evolution experiment. Variants in only the coding region are shown, excluding intergenic regions and hypothetical proteins. For variants that span more than a single nucleotide, the variant frequency may appear as a range to indicate the minimum/maximum variant frequency over that range.

| **Sample** | **Gene** | **Product** | **Change** | **CDS position** | **Protein effect** | **Variant frequency** |
| --- | --- | --- | --- | --- | --- | --- |
| **GROUP 1 REPRESENTATIVE - UNTREATED** | cysG | Siroheme synthase | G -> C | 263 | Substitution | 15.20% |
|  | N/A | Lipase | G -> C | 1359 | Substitution | 15.10% |
|  | macB | Macrolide export ATP-binding/permease protein MacB | C -> G | 1790 | Substitution | 14.40% |
|  | macB | Macrolide export ATP-binding/permease protein MacB | C -> A | 1799 | Substitution | 13.00% |
|  | N/A | Putative multidrug export ATP-binding/permease protein | T -> C | 904 | Substitution | 12.60% |
|  | hexR | HTH-type transcriptional regulator HexR | T -> C | 693 | None | 12.50% |
|  | hexR | HTH-type transcriptional regulator HexR | A -> C | 689 | Substitution | 11.90% |
|  | potH | Putrescine transport system permease protein PotH | G -> T | 145 | Substitution | 11.30% |
|  | N/A | putative methyltransferase | C -> A | 436 | Substitution | 11.30% |
|  | N/A | putative methyltransferase | G -> A | 434 | Substitution | 11.30% |
|  | potH | Putrescine transport system permease protein PotH | T -> A | 155 | Substitution | 10.70% |
|  | N/A | putative methyltransferase | A -> T | 446 | Substitution | 10.50% |
|  | N/A | putative methyltransferase | C -> A | 444 | Substitution | 10.30% |
|  | mcpG | Methyl-accepting chemotaxis protein McpG | T -> G | 1763 | Substitution | 10.10% |
| **GROUP 1 REPRESENTATIVE - TREATED** | rpoA | DNA-directed RNA polymerase subunit alpha | G -> C | 901 | Substitution | 71.80% |
|  | tdh | L-threonine 3-dehydrogenase | CGGCGCCAGCACATCG -> TCTTTTTCAGAGTTTC | 693 | Truncation | 38.8% -> 45.8% |
|  | tdh | L-threonine 3-dehydrogenase | CA -> GG | 686 | Substitution | 40.0% -> 41.9% |
|  | tdh | L-threonine 3-dehydrogenase | CTGCCGC -> TCAGAAA | 710 | Substitution | 35.9% -> 40.0% |
|  | tdh | L-threonine 3-dehydrogenase | CGACA -> TGGTC | 680 | Substitution | 36.7% -> 39.1% |
|  | tdh | L-threonine 3-dehydrogenase | CTGCGG -> GAAGTA | 673 | Substitution | 33.1% -> 35.4% |
|  | tdh | L-threonine 3-dehydrogenase | G -> A | 718 | None | 34.10% |
|  | tdh | L-threonine 3-dehydrogenase | CC -> TG | 667 | Substitution | 31.1% -> 31.7% |
|  | tdh | L-threonine 3-dehydrogenase | CAGAA -> TTTTG | 722 | Substitution | 28.9% -> 31.5% |
|  | tdh | L-threonine 3-dehydrogenase | AAC -> TTT | 662 | Substitution | 29.8% -> 30.2% |
|  | tdh | L-threonine 3-dehydrogenase | AT -> CC | 659 | Substitution | 26.7% -> 27.2% |
|  | tdh | L-threonine 3-dehydrogenase | CAGCCC -> GTCGCG | 730 | Substitution | 21.4% -> 24.6% |
|  | preT | NAD-dependent dihydropyrimidine dehydrogenase subunit PreT | T -> G | 29 | Substitution | 24.30% |
|  | tdh | L-threonine 3-dehydrogenase | A -> GC | 656 | Frame Shift | 18.6% -> 21.1% |
|  | tdh | L-threonine 3-dehydrogenase | C -> T | 654 | None | 19.00% |
|  | N/A | Lipase | G -> C | 1392 | Substitution | 17.10% |
|  | tdh | L-threonine 3-dehydrogenase | GTC -> CTA | 646 | Truncation | 16.4% -> 17.0% |
|  | tdh | L-threonine 3-dehydrogenase | CCTT -> GTGG | 737 | Substitution | 16.0% -> 16.3% |
|  | mcpG | Methyl-accepting chemotaxis protein McpG | T -> G | 1763 | Substitution | 15.70% |
|  | tdh | L-threonine 3-dehydrogenase | ACGCCGCG -> CGCAACTT | 637 | Substitution | 10.5% -> 15.4% |
|  | N/A | Putative multidrug export ATP-binding/permease protein | T -> C | 904 | Substitution | 15.10% |
|  | macB | Macrolide export ATP-binding/permease protein MacB | A -> T | 1805 | Substitution | 14.60% |
|  | mcpG | Methyl-accepting chemotaxis protein McpG | A -> G | 1766 | Substitution | 14.20% |
|  | macB | Macrolide export ATP-binding/permease protein MacB | C -> A | 1799 | Substitution | 13.60% |
|  | N/A | putative methyltransferase | A -> T | 446 | Substitution | 13.40% |
|  | potH | Putrescine transport system permease protein PotH | A -> T | 141 | Substitution | 13.30% |
|  | macB | Macrolide export ATP-binding/permease protein MacB | C -> G | 1790 | Substitution | 12.60% |
|  | pnp | Polyribonucleotide nucleotidyltransferase | T -> A | 1179 | None | 12.40% |
|  | fadE | Acyl-coenzyme A dehydrogenase | T -> C | 134 | Substitution | 12.40% |
|  | cdsA | Phosphatidate cytidylyltransferase | C -> G | 659 | Substitution | 11.80% |
|  | potH | Putrescine transport system permease protein PotH | G -> T | 148 | Substitution | 11.70% |
|  | mcpG | Methyl-accepting chemotaxis protein McpG | A -> T | 1754 | Substitution | 11.60% |
|  | tdh | L-threonine 3-dehydrogenase | CC -> TG | 742 | Substitution | 10.5% -> 11.4% |
|  | amtB | Ammonia channel | T -> C | 1199 | Substitution | 11.40% |
|  | N/A | putative methyltransferase | CAC -> AAA | 436 | Substitution | 10.8% -> 11.3% |
|  | macB | Macrolide export ATP-binding/permease protein MacB | C -> T | 1797 | None | 10.90% |
|  | wzxE | Lipid III flippase | C -> A | 390 | None | 10.90% |
|  | pnp | Polyribonucleotide nucleotidyltransferase | T -> A | 1176 | None | 10.80% |
|  | N/A | putative methyltransferase | C -> A | 432 | Substitution | 10.50% |
|  | mcpG | Methyl-accepting chemotaxis protein McpG | A -> G | 1760 | Substitution | 10.30% |
|  | N/A | putative methyltransferase | GTC -> ATA | 439 | Substitution | 10.1% -> 10.2% |
|  | aroF | Phospho-2-dehydro-3-deoxyheptonate aldolase, Tyr-sensitive | T -> A | 995 | Substitution | 10.10% |
|  | N/A | putative methyltransferase | CA -> TT | 442 | Substitution | 10.00% |
| **GROUP 2 REPRESENTATIVE - UNTREATED** | holC | DNA polymerase III subunit chi | G -> A | 50 | Substitution | 21.80% |
|  | cysG | Siroheme synthase | G -> C | 298 | Substitution | 20.50% |
|  | cysG | Siroheme synthase | G -> C | 307 | Substitution | 18.90% |
|  | preT | NAD-dependent dihydropyrimidine dehydrogenase subunit PreT | A -> G | 23 | Substitution | 17.80% |
|  | N/A | Lipase | G -> C | 1359 | Substitution | 15.00% |
|  | macB | Macrolide export ATP-binding/permease protein MacB | C -> A | 1799 | Substitution | 14.80% |
|  | N/A | Lipase | A -> C | 1382 | Substitution | 14.40% |
|  | N/A | Putative multidrug export ATP-binding/permease protein | T -> C | 904 | Substitution | 14.20% |
|  | macB | Macrolide export ATP-binding/permease protein MacB | A -> T | 1805 | Substitution | 14.20% |
|  | N/A | Lipase | G -> C | 1419 | Substitution | 14.20% |
|  | potH | Putrescine transport system permease protein PotH | A -> C | 164 | Substitution | 13.00% |
|  | potH | Putrescine transport system permease protein PotH | T -> G | 159 | None | 12.20% |
|  | macB | Macrolide export ATP-binding/permease protein MacB | CA -> TG | 1796 | Substitution | 11.9% -> 12.1% |
|  | N/A | putative methyltransferase | TCC -> AAA | 438 | Substitution | 11.1% -> 11.3% |
|  | potH | Putrescine transport system permease protein PotH | C -> G | 160 | Substitution | 11.30% |
|  | N/A | putative methyltransferase | A -> T | 442 | Substitution | 11.30% |
|  | mcpG | Methyl-accepting chemotaxis protein McpG | A -> G | 1766 | Substitution | 11.10% |
|  | potH | Putrescine transport system permease protein PotH | A -> G | 154 | Substitution | 10.80% |
|  | N/A | putative methyltransferase | C -> A | 435 | None | 10.60% |
|  | macB | Macrolide export ATP-binding/permease protein MacB | C -> G | 1790 | Substitution | 10.50% |
|  | mcpG | Methyl-accepting chemotaxis protein McpG | T -> G | 1763 | Substitution | 10.30% |
| **GROUP 2 REPRESENTATIVE - TREATED** | fliA | RNA polymerase sigma factor FliA | T -> G | 659 | Substitution | 100.00% |
|  | tdh | L-threonine 3-dehydrogenase | A -> G | 28 | Substitution | 99.40% |
|  | roxA | 50S ribosomal protein L16 3-hydroxylase | T -> C | 320 | Substitution | 57.60% |
|  | roxA | 50S ribosomal protein L16 3-hydroxylase | TG -> AT | 322 | Substitution | 56.7% -> 57.1% |
|  | roxA | 50S ribosomal protein L16 3-hydroxylase | A -> C | 325 | Substitution | 54.60% |
|  | roxA | 50S ribosomal protein L16 3-hydroxylase | GCG -> CA | 328 | Frame Shift | 35.0% -> 36.9% |
|  | roxA | 50S ribosomal protein L16 3-hydroxylase | AG -> TA | 334 | Truncation | 36.4% -> 36.8% |
|  | roxA | 50S ribosomal protein L16 3-hydroxylase | GAA -> CTT | 331 | Substitution | 35.5% -> 35.8% |
|  | roxA | 50S ribosomal protein L16 3-hydroxylase | CAGTT -> GGAAA | 311 | Substitution | 33.1% -> 34.1% |
|  | roxA | 50S ribosomal protein L16 3-hydroxylase | GCCACTTCCGG -> CTGAAAAAGAC | 298 | Substitution | 24.6% -> 31.0% |
|  | roxA | 50S ribosomal protein L16 3-hydroxylase | GCT -> ACA | 340 | Substitution | 28.8% -> 30.4% |
|  | roxA | 50S ribosomal protein L16 3-hydroxylase | CCA -> AAT | 343 | Substitution | 27.0% -> 27.9% |
|  | roxA | 50S ribosomal protein L16 3-hydroxylase | CG -> GA | 346 | Substitution | 26.1% -> 26.4% |
|  | roxA | 50S ribosomal protein L16 3-hydroxylase | GAT -> CGG | 349 | Substitution | 21.1% -> 22.2% |
|  | roxA | 50S ribosomal protein L16 3-hydroxylase | GT -> TG | 353 | Substitution | 18.5% -> 18.7% |
|  | N/A | Lipase | A -> C | 1382 | Substitution | 18.50% |
|  | roxA | 50S ribosomal protein L16 3-hydroxylase | ATC -> GTA | 355 | Substitution | 15.7% -> 17.6% |
|  | nodD2 | Nodulation protein D 2 | A -> C | 899 | Substitution | 16.80% |
|  | N/A | Putative multidrug export ATP-binding/permease protein | T -> C | 904 | Substitution | 15.80% |
|  | macB | Macrolide export ATP-binding/permease protein MacB | C -> G | 1790 | Substitution | 15.60% |
|  | macB | Macrolide export ATP-binding/permease protein MacB | A -> T | 1805 | Substitution | 15.20% |
|  | N/A | 6-phosphogluconate dehydrogenase, NAD(+)-dependent, decarboxylating | T -> G | 212 | Substitution | 15.10% |
|  | pnp | Polyribonucleotide nucleotidyltransferase | A -> C | 1169 | Substitution | 14.40% |
|  | roxA | 50S ribosomal protein L16 3-hydroxylase | GCG -> TCA | 328 | Truncation | 13.9% -> 14.2% |
|  | preT | NAD-dependent dihydropyrimidine dehydrogenase subunit PreT | T -> G | 29 | Substitution | 14.20% |
|  | roxA | 50S ribosomal protein L16 3-hydroxylase | CAC -> GTG | 358 | Substitution | 13.0% -> 14.0% |
|  | macB | Macrolide export ATP-binding/permease protein MacB | C -> A | 1799 | Substitution | 13.80% |
|  | roxA | 50S ribosomal protein L16 3-hydroxylase | ACGAACTGATCGACCGCT -> TTCTGATTTGGCAAAATA | 279 | Substitution | 9.5% -> 13.7% |
|  | potH | Putrescine transport system permease protein PotH | A -> C | 164 | Substitution | 13.50% |
|  | roxA | 50S ribosomal protein L16 3-hydroxylase | GAA -> ACT | 331 | Substitution | 12.5% -> 13.3% |
|  | N/A | putative methyltransferase | C -> A | 438 | None | 13.20% |
|  | roxA | 50S ribosomal protein L16 3-hydroxylase | CAT -> TAG | 361 | Substitution | 11.1% -> 12.8% |
|  | roxA | 50S ribosomal protein L16 3-hydroxylase | C -> T | 368 | Substitution | 12.70% |
|  | hexR | HTH-type transcriptional regulator HexR | A -> C | 683 | Substitution | 12.00% |
|  | N/A | putative methyltransferase | C -> A | 435 | None | 11.80% |
|  | roxA | 50S ribosomal protein L16 3-hydroxylase | CC -> AA | 344 | Substitution | 11.6% -> 11.7% |
|  | potH | Putrescine transport system permease protein PotH | A -> G | 154 | Substitution | 11.60% |
|  | roxA | 50S ribosomal protein L16 3-hydroxylase | AT -> CG | 364 | Substitution | 11.2% -> 11.5% |
|  | potH | Putrescine transport system permease protein PotH | A -> T | 141 | Substitution | 11.40% |
|  | potH | Putrescine transport system permease protein PotH | A -> T | 146 | Substitution | 11.30% |
|  | roxA | 50S ribosomal protein L16 3-hydroxylase | CAC -> GGT | 358 | Substitution | 11.1% -> 11.2% |
|  | roxA | 50S ribosomal protein L16 3-hydroxylase | ATC -> GGT | 355 | Substitution | 11.1% -> 11.2% |
|  | roxA | 50S ribosomal protein L16 3-hydroxylase | GTC -> ATG | 352 | Substitution | 11.1% -> 11.2% |
|  | roxA | 50S ribosomal protein L16 3-hydroxylase | AT -> TA | 361 | Truncation | 11.0% -> 11.2% |
|  | ycfH | putative metal-dependent hydrolase YcfH | A -> C | 731 | Substitution | 11.20% |
|  | roxA | 50S ribosomal protein L16 3-hydroxylase | GAT -> CCG | 349 | Substitution | 11.0% -> 11.1% |
|  | mcpG | Methyl-accepting chemotaxis protein McpG | A -> G | 1766 | Substitution | 11.10% |
|  | roxA | 50S ribosomal protein L16 3-hydroxylase | AG -> CT | 334 | Substitution | 10.3% -> 11.0% |
|  | mcpG | Methyl-accepting chemotaxis protein McpG | T -> G | 1763 | Substitution | 11.00% |
|  | potH | Putrescine transport system permease protein PotH | C -> G | 160 | Substitution | 10.80% |
|  | roxA | 50S ribosomal protein L16 3-hydroxylase | C -> G | 347 | Substitution | 10.80% |
|  | amtB | Ammonia channel | T -> C | 1193 | Substitution | 10.50% |
|  | pgl | 6-phosphogluconolactonase | T -> C | 752 | Substitution | 10.50% |
|  | hexR | HTH-type transcriptional regulator HexR | T -> C | 693 | None | 10.40% |
|  | cfiB | 2-oxoglutarate carboxylase small subunit | G -> C | 1124 | Substitution | 10.10% |
|  | potH | Putrescine transport system permease protein PotH | C -> T | 144 | None | 10.10% |
|  | proP | Proline/betaine transporter | G -> C | 1490 | Substitution | 10.10% |
|  | proP | Proline/betaine transporter | G -> T | 1486 | Substitution | 10.10% |
|  | cfiB | 2-oxoglutarate carboxylase small subunit | A -> C | 1130 | Substitution | 10.00% |
|  | ptrB | Protease 2 | T -> C | 453 | None | 10.00% |
| **GROUP 3 REPRESENTATIVE - UNTREATED** | motA | Motility protein A | C -> G | 328 | Substitution | 33.70% |
|  | motA | Motility protein A | AAA -> TGG | 323 | Substitution | 28.4% -> 30.8% |
|  | motA | Motility protein A | G -> T | 320 | Substitution | 27.30% |
|  | motA | Motility protein A | GCTGGCGGCGGCGTC -> AAGTAGCCATGTATT | 304 | Substitution | 20.3% -> 21.0% |
|  | motA | Motility protein A | T -> C | 302 | Substitution | 20.30% |
|  | motA | Motility protein A | GAT -> CCG | 298 | Substitution | 18.6% -> 19.5% |
|  | motA | Motility protein A | TCGG -> CATA | 293 | Substitution | 16.1% -> 17.1% |
|  | preT | NAD-dependent dihydropyrimidine dehydrogenase subunit PreT | A -> G | 23 | Substitution | 15.90% |
|  | melD | Melibiose/raffinose/stachyose import permease protein MelD | T -> G | 776 | Substitution | 14.70% |
|  | N/A | putative methyltransferase | T -> A | 440 | Substitution | 14.30% |
|  | macB | Macrolide export ATP-binding/permease protein MacB | A -> G | 1796 | Substitution | 13.60% |
|  | preT | NAD-dependent dihydropyrimidine dehydrogenase subunit PreT | T -> G | 29 | Substitution | 13.60% |
|  | motA | Motility protein A | T -> A | 290 | Substitution | 13.20% |
|  | motA | Motility protein A | GAT -> CTA | 286 | Truncation | 13.0% -> 13.1% |
|  | motA | Motility protein A | GC -> CG | 283 | Substitution | 10.1% -> 11.8% |
|  | potH | Putrescine transport system permease protein PotH | C -> G | 160 | Substitution | 11.60% |
|  | cat | Catalase | ATCG -> CTGT | 373 | Substitution | 11.1% -> 11.3% |
|  | cat | Catalase | T -> C | 381 | None | 11.20% |
|  | cat | Catalase | A -> G | 396 | None | 11.10% |
|  | mcpG | Methyl-accepting chemotaxis protein McpG | T -> G | 1763 | Substitution | 11.00% |
|  | macB | Macrolide export ATP-binding/permease protein MacB | C -> A | 1799 | Substitution | 11.00% |
|  | potH | Putrescine transport system permease protein PotH | TA -> AC | 163 | Substitution | 10.7% -> 10.8% |
|  | macB | Macrolide export ATP-binding/permease protein MacB | C -> G | 1790 | Substitution | 10.80% |
|  | cat | Catalase | ACA -> TTC | 388 | Substitution | 10.6% -> 10.7% |
|  | cat | Catalase | T -> C | 405 | None | 10.70% |
|  | N/A | putative methyltransferase | A -> T | 446 | Substitution | 10.70% |
|  | cat | Catalase | G -> A | 393 | None | 10.60% |
|  | motA | Motility protein A | CGGCCGGGTA -> TCAGAGTTTC | 334 | Substitution | 9.7% -> 10.5% |
|  | cat | Catalase | A -> G | 356 | Substitution | 10.50% |
|  | cat | Catalase | C -> G | 412 | Substitution | 10.50% |
|  | cat | Catalase | TTTG -> AGTC | 339 | Substitution | 10.2% -> 10.4% |
|  | cat | Catalase | T -> C | 333 | None | 10.20% |
|  | cat | Catalase | T -> A | 415 | Substitution | 10.20% |
|  | potH | Putrescine transport system permease protein PotH | A -> T | 141 | Substitution | 10.10% |
|  | cat | Catalase | C -> T | 330 | None | 10.10% |
|  | cat | Catalase | C -> T | 327 | None | 10.00% |
| **GROUP 3 REPRESENTATIVE - TREATED** | gacA | Response regulator GacA | -TCAGGCGCTGGCGTCCACCATGCCGTGG | 558 | Extension | 77.2% -> 81.3% |
|  | uvrC | UvrABC system protein C | -TCAT | 1 | Start Codon Loss | 77.7% -> 78.1% |
|  | prmC | Release factor glutamine methyltransferase | (GCGG)2 -> (GCGG)3 | 323 | Frame Shift | 29.30% |
|  | hprA | Glycerate dehydrogenase | G -> T | 316 | Substitution | 29.00% |
|  | preT | NAD-dependent dihydropyrimidine dehydrogenase subunit PreT | T -> G | 29 | Substitution | 22.90% |
|  | preT | NAD-dependent dihydropyrimidine dehydrogenase subunit PreT | A -> G | 36 | None | 21.90% |
|  | N/A | putative methyltransferase | A -> G | 455 | Substitution | 21.50% |
|  | N/A | Lipase | G -> C | 1392 | Substitution | 20.90% |
|  | N/A | Lipase | A -> C | 1400 | Substitution | 20.60% |
|  | preT | NAD-dependent dihydropyrimidine dehydrogenase subunit PreT | A -> G | 23 | Substitution | 17.40% |
|  | macB | Macrolide export ATP-binding/permease protein MacB | A -> G | 1796 | Substitution | 17.10% |
|  | macB | Macrolide export ATP-binding/permease protein MacB | C -> A | 1799 | Substitution | 16.70% |
|  | N/A | putative methyltransferase | C -> A | 435 | None | 14.50% |
|  | N/A | putative methyltransferase | C -> A | 444 | Substitution | 13.50% |
|  | N/A | Putative multidrug export ATP-binding/permease protein | T -> C | 904 | Substitution | 12.80% |
|  | potH | Putrescine transport system permease protein PotH | T -> G | 159 | None | 11.50% |
|  | macB | Macrolide export ATP-binding/permease protein MacB | C -> G | 1790 | Substitution | 11.30% |
|  | potH | Putrescine transport system permease protein PotH | A -> C | 164 | Substitution | 11.10% |
|  | potH | Putrescine transport system permease protein PotH | A -> G | 154 | Substitution | 10.60% |
